# The Contribution of Serum Complement Component 3 Levels to 90-Day Mortality in Living Donor Liver Transplantation

**DOI:** 10.3389/fimmu.2021.652677

**Published:** 2021-07-19

**Authors:** Saeko Fukui, Masaaki Hidaka, Shoichi Fukui, Shimpei Morimoto, Takanobu Hara, Akihiko Soyama, Tomohiko Adachi, Hajime Matsushima, Takayuki Tanaka, Mai Fuchigami, Hiroo Hasegawa, Katsunori Yanagihara, Susumu Eguchi

**Affiliations:** ^1^ Department of Surgery, Nagasaki University Graduate School of Biomedical Sciences, Nagasaki, Japan; ^2^ Program in Cellular and Molecular Medicine, Boston Children’s Hospital, Boston, MA, United States; ^3^ Innovation Platform & Office for Precision Medicine, Nagasaki University Graduate School of Biomedical Sciences, Nagasaki, Japan; ^4^ Department of Laboratory Medicine, Nagasaki University Graduate School of Biomedical Sciences, Nagasaki, Japan

**Keywords:** C3, C4, immunoglobulin G, posttransplant infection, leukocyte populations

## Abstract

The contributions of the complement system have been elucidated in the process of solid organ transplantation, including kidney transplantation. However, the role of complement in liver transplantation is unknown. We sought to elucidate the time-dependent changes of peritransplantational serum complement levels and the relationships with posttransplant outcomes and other immunological biomarkers. We enrolled 82 patients who underwent living-related donor liver transplantation (LDLT). Nine patients (11%) died within 90 days after LDLT (non-survivors). The following immunomarkers were collected preoperatively and at 1, 2, and 4 week(s) after LDLT: serum C3, C4, immunoglobulin G (IgG), and peripheral blood leukocyte populations characterized by CD3, CD4, CD8, CD16, CD19, CD20, CD22, and CD56. Consequently, C3 and C4 increased time-dependently after LDLT. Preoperatively, C3 was negatively correlated with the MELD score, Child–Pugh score, CD16-positive leukocyte percentage, and the CD56-positive leukocyte percentage. Non-survivors had lower levels of C3 at 2 weeks in comparison to survivors (median [interquartile range]: 56 [49-70] mg/dL *vs.* 88 [71-116] mg/dL, p=0.0059). When the cutoff value of C3 at 2 weeks to distinguish non-survivors was set to 71 mg/dL, the sensitivity, specificity, and area under the ROC curve were 87.5%, 75.0%, and 0.80, respectively. A principal component analysis showed an inverse relationship between the C3 and C4 levels and the percentage of CD8-, CD16-, and CD56-positive leukocytes at 1 and 2 week(s). All non-survivors were included in the cluster that showed higher percentages of CD8-, CD16-, and CD56-positive leukocytes at 2 weeks. In conclusion, we demonstrated the relationship between complement, outcomes, and other immunomarkers in LDLT and suggested the usefulness of C3 at 2 weeks after LDLT in distinguishing the mortality.

## Introduction

Complement has become accepted as a possible efficient biomarker associated with the outcomes of solid organ transplantation (SOT), including kidney transplantation (1). New therapeutic strategies are used to control complement, in addition to conventional immunosuppressive therapies targeting cellular immunity (2, 3). At the same time, the need to understand the role of complement in SOT has been expanding. The complement system works as a part of the innate immune system, which is responsible for the initiation of non-self-recognition that eventually leads to the establishment of adaptive immunity (4). The activation of complement cascades through three major pathways enables the effective removal of pathogens by the mononuclear phagocyte system or destruction by the membrane attack complex, which is composed of complement components C5b, C6, C7, C8, C9. Activated components also promote an inflammatory response and the elimination of foreign material by the adaptive immune system (4). However, complement levels are not routinely assessed in liver transplant recipients, despite the important role in immunity (5). The perioperative kinetics of complement, the correlation with other humoral and cellular markers, and the relationship with posttransplant outcomes remain unclear.

In the area of liver transplantation (LT), limited studies have attempted to comprehensively analyze complement with other markers of cellular immunity, although the liver is the major site for the synthesis of complement components. Carbone et al. (6) suggested that lower pretransplantation levels of serum C3 and higher immunoglobulin G (IgG) levels might be useful for predicting the risk of infection after LT, although individual lymphocyte subsets—characterized by CD3, CD4, CD8, CD16, CD56, or CD19—might not (6). Fernández-Ruiz et al. (7) demonstrated that the lymphocyte count at baseline predicted the incidence of infection after LT (7), and Nierenberg et al. reported that pretransplant lymphopenia was a strong predictor of cytomegalovirus (CMV) infection after LT (8). Despite these reports, the relationships between humoral immunomarkers and leukocyte subpopulations in LT have not been elucidated.

We sought to comprehensively observe the serum complement levels at pretransplantation and posttransplantation time points, along with other immunological markers, and to clarify the time-dependent changes and correlations among markers at each time point. In addition, we sought to assess the relationship between complement components and posttransplantation outcomes, including mortality and the development of infection. We assumed these results would contribute to the elucidation of the clinical meaning of complement in LT and give suggestions for new therapeutic strategies involving the complement system.

## Patients And Methods

### Patients

We reviewed the cases of all patients who underwent living-related donor liver transplantation (LDLT) at Nagasaki University Hospital from January 1, 2013 to February 15, 2018. Four cases without detailed charts and one case in which the patient died due to intraoperative uncontrolled bleeding were excluded and we finally enrolled 82 patients who underwent LDLT in this study. This study was performed in accordance with the Declaration of Helsinki and was approved by the Institutional Review Board of Nagasaki University Hospital (registration number: 18101501). Written consent forms were obtained from some of the patients, an opt-out strategy was used to obtain consent from the rest of the patients.

### Surgical Procedures and Perioperative Management

We selected the left lobe graft with a middle hepatic vein when the ratio of the graft volume to the recipient standard liver volume (GV/SLV) was >30%. The right lobe graft was an alternative if the left lobe was not feasible for donation. The ratio was calculated from the results of a volumetric study using computed tomography. Arterial reconstruction was carried out under a microscope using end-to-end anastomosis with interrupted suture techniques (9). Duct-to-duct (D-D) anastomosis was performed for biliary reconstruction, except in patients with primary sclerosing cholangitis. A biliary splint (2 mm, vinyl chloride tube) was placed beyond the site of anastomosis and the splint was externalized through the upper edge of the duodenum with a Witzel-type fistula. The splint was removed approximately 3 months after LDLT using a two-step protocol (10): the tube was withdrawn under X-ray control to the most peripheral part of the tract established by Witzel’s canalization. On the following day, after confirming the absence of bile leakage and peritonitis, the tube was completely removed. Antimicrobial prophylaxis consisted of cefotaxime (4 g/day) and ampicillin (4 g/day). These medications were initiated 30 min before laparotomy and continued up to 48 hours after LDLT. If the patient had pretransplant infection within 2 weeks prior to LDLT, their antimicrobial therapy was continued perioperatively. Prophylactic valganciclovir (10 mg/kg/day) was given for 14 days when the donor was positive but the recipient was negative for anticytomegalovirus immunoglobulin G; trimethoprim–sulfamethoxazole (1 g/day) was administered for one month as prophylaxis against *Pneumocystis jirovecii* pneumonia. Early nutritional therapy was given to all recipients by tube jejunostomy or nasojejunal feeding tube from the day of LDLT to the day on which the recipient could eat sufficiently.

### Immunosuppression Therapy

The standard immunosuppression regimen consisted of tacrolimus and corticosteroids. The target trough levels of tacrolimus were 10–15 ng/mL until 1 month after surgery and were tapered to ≤10 ng/ml thereafter. Methylprednisolone (1 g, intravenously) was administered just before reperfusion during surgery. During the postoperative period, we administered methylprednisolone at a dose of 0.5 mg/kg intravenously, four times a day, for the first 3 postoperative days, followed by 0.5 mg/kg, twice a day, for the next 3 days. Thereafter, methylprednisolone was switched to oral prednisolone (0.5 mg/kg, once a day) at 7 days after transplantation, and oral prednisolone was discontinued by 3 months after LDLT. In ABO-incompatible LDLT cases, we used rituximab (375 mg/m^2^) as an induction 10–14 days before LDLT and added mycophenolate mofetil (MMF) after LDLT. For patients who have renal dysfunction, trough levels of tacrolimus were intentionally kept lower by the administration of other immunosuppressants, including MMF and/or basiliximab (11).

### Data Collection and Definition

We collected the demographic and clinical characteristics and laboratory data at the preoperative evaluations, 1, 2, and 4 week(s) after LDLT from the patients’ medical records. We collected the records of infectious episodes requiring antibiotic treatment, cytomegalovirus infection, bacteremia, and death as outcomes. The use of antibiotics was at the discretion of the attending surgeon based on clinical manifestations, culture results, and laboratory data, with advice by infectious disease specialists. Cytomegalovirus infection was defined as detectable pp65-positive cells by the antigenemia method (C7-HRP) and the necessity of the treatment with ganciclovir or foscavir. Bacteremia was defined as positive blood cultures without contamination. Early allograft dysfunction was defined as the presence of one or more of the following laboratory analyses: bilirubin ≥10mg/dL on day 7, international normalized ratio ≥1.6 on day 7, and alanine or aspartate aminotransferases >2000 IU/L within the first 7 days (12). Acute cellular rejection was diagnosed by histopathological examination of biopsy specimens according to Banff schema for grading liver allograft rejection (13).

### C3, C4, IgG Measurement

This study was performed using preserved patient sera. Patient sera were obtained just before operations and at 1, 2, and 4 weeks after LDLT. Patient blood was collected in serum separator tubes and centrifuged at 3000 rpm for 5 minutes at room temperature. Sera were collected immediately afterwards and stored at -80°C without thawing before the use. Serum C3 and C4 was measured with a commercial turbidimetric immunoassay (N‐assay TIA C3‐SH, C4‐SH, respectively; Nittobo Medical, Tokyo, Japan). The inter-assay and intra-assay variations were 0.79% and 1.18%, respectively. The normal reference ranges were as follows: C3, 86-160 mg/dL; C4, 17-45 mg/dL; IgG, 870-1,700 mg/dL.

### The Flow Cytometric Analysis of Peripheral Blood Leukocyte Populations

The flow cytometric analysis (FCM) of leukocyte populations were performed by three-color fluorescence methodology. Peripheral blood cells were stained with antibodies to CD45/PerCP (BD Biosciences, CA, USA), CD3/PE (BD), CD4/FITC (BD), CD8/PE (BD), CD16/FITC (Beckman Coulter, CA, USA), CD56/PE (BD), CD19/FITC (BD), CD20/PE (BD), and CD22/PE (Dako, Tokyo, Japan). After gating lymphocytes by CD45/side-scatter, expression profiling of CD markers was performed using a FACS Canto II (BD) and was analyzed using the BD FACS Diva software program (ver.6.1.2; BD).

### Statistical Analysis

Bonferroni correction was applied for time-dependent changes in serum C3, C4, and IgG. Other p-values were displayed without adjustment of multiple-testing because the present study was performed as exploratory research. Categorical variables are reported as frequencies and continuous variables are reported as the median and interquartile range (IQR). Associations between variables were assessed using Fisher’s exact test for categorical variables and the Wilcoxon rank-sum test for continuous variables. The Wilcoxon signed-rank test was used to assess differences in serum levels of C3, C4, and IgG between 0, 1, 2, and 4 week(s) after LDLT. Correlation was analyzed using Spearman’s rank correlation coefficient.

k-means clustering (14) was performed for the serum C3, C4, IgG levels and the peripheral leukocyte populations, and yielded three clusters based on scree plots. In addition to the clustering analysis, we sought a principal coordinate to describe the patients’ immunological status using multidimensional scaling *via* a principal component analysis (PCA). The cumulative incidence of CMV infection and the survival function of the days from LDLT were estimated using the Kaplan-Meier method. The null hypothesis of differences between the survival functions was tested by a log-rank test.

In order to evaluate the changes in the serum C3 levels, we created a “C3 ratio” which is defined as the C3 level at 2 weeks divided by the C3 level at 1 week after LDLT. The odds ratio of C3 ratio of ≤1.09 for 90-day mortality was estimated as the association of a low C3 ratio in patients who had characteristics that caused high C3 ratio, using logistic regression with balancing weight (15, 16). Designing to adjust for the imbalance in clinical characteristics that were thought to affect both C3 ratio and 90-day mortality, the balancing weight consisted of linear combination of episodes of infection by day 7 and early allograft dysfunction at 1 week; acute cellular rejection at 2 weeks was assumable to be included as well, however, this factor was adjusted as a covariate in the logistic regression model since the imbalance in this factor was not mitigated by including into the balancing weight. The balance in these variables after weighting was assessed by the standardized mean differences displayed as a line plot. (**Supplementary Figure 1**). The Harrell’s C-statistic of the propensity score was 0.837 (95% confidence interval: 0.736, 0.938). The density and distribution of the propensity score were shown as a density plot and a step function of cumulative distribution, respectively (**Supplementary Figure 2**). The standard error of regression coefficient was estimated by a Horvitz-Thompson estimator (17, 18). The R source code for the analysis in this paragraph is available from GitHub (https://github.com/mrmtshmp/sampleDesign_20.01).

All statistical analyses were conducted under the R environment v. 4.0.0 (19) using relevant packages [tidyverse (20), ggplot2 (21), corrplot (22), plotROC (23), survival (24), survminer (25), survey (16), factoextra (26), RColorBrewer (27), tableone (28)].

## Results

### Patient Characteristics

The demographic, clinical, and laboratory characteristics of the patients at the preoperative examination and the C3, C4, and IgG levels at 1, 2, and 4 week(s) after LDLT, and the outcomes are summarized in **Table 1**. Our cohort was predominantly male (54.9%). The median age was 59 years. The median Child–Pugh score and median model for end-stage liver disease (MELD) score were 11 and 17, respectively. Thirty patients (37%) had hepatocellular carcinoma (HCC).

**Table 1:** Clinical characteristics of all patients, survivors, and non-survivors.

Clinical characteristics, median (IQR) or n (%)	All patients	Non-survivors (n = 9)	Survivors (n = 73)	Number*	P value
Age (years)	59 (53–64)	63 (59–65)	59 (52–63)	9:73	0.1239
Sex (male)	45 (55)	3 (33)	42 (58)	9:73	0.2871
Hight (m)	1.62 (1.53-1.69)	1.52 (1.50-1.68)	1.62 (1.53-1.69)	9:73	0.1120
Body weight (kg)	61.6 (53.2-72.5)	61.9 (52.8-71.0)	61.5 (54.4-72.5)	9:73	0.8704
Body mass index (kg/m^2^)	23.5 (21.5-26.6)	22.9 (21.8-27.4)	23.6 (21.5-26.5)	9:73	0.6671
Child–Pugh score	11 (9–12)	11 (10–12)	11 (9–12)	9:73	0.6417
MELD score at transplantation	17 (13–23)	16 (14–27)	17 (13–22)	9:73	0.4713
GW/SLV	42 (34–52)	39 (34–45)	42 (34–52)	9:73	0.2412
Donor age (years)	38 (30–47)	42 (39–54)	36 (28–44)	9:73	0.0250**
Donor sex (male)	43 (52)	6 (67)	37 (51)	9:73	0.4874
Cold ischemic time (minutes)	86 (60–97)	70 (61–88)	86 (60–97)	9:73	0.5186
Operative time (minutes)	755 (688–859)	822 (740–877)	749 (686–851)	9:73	0.2991
Blood loss (mL)	5,800 (3,600–10,000)	10,000 (4,900–16,600)	5,600 (3,600–8,900)	9:73	0.1843
Splenectomy	37 (45)	5 (56)	32 (44)	9:73	0.7247
Other immunosuppressants	61 (74)	8 (89)	53 (73)	9:73	0.4354
DD-reconstruction	75 (91)	9 (100)	66 (90)	9:73	1.0000
HCC	30 (37)	4 (44)	26 (36)	9:73	0.7179
HBV positive	7 (9)	0 (0)	7 (10)	9:73	1.0000
HCV positive	26 (32)	4 (44)	22 (30)	9:73	0.4547
Alcoholic Cirrhosis	19 (23)	1 (11)	18 (25)	9:73	0.6772
Nonalcoholic fatty liver disease	6 (7)	1 (11)	5 (7)	9:73	0.5139
Preoperative ICU	9 (11)	3 (33)	6 (8)	9:73	0.0557
Child-Pugh C	55 (67)	7 (78)	48 (66)	9:73	0.7106
ABO incompatible	21 (26)	2 (22)	19 (26)	9:73	1.0000
Preoperative bacteremia	5 (6)	1 (11)	4 (5)	9:73	0.4495
HLA mismatch	3 (2–3)	3 (3–4)	3 (2–3)	9:73	0.2346
White blood cells (/μL)	4,600 (3,100–6,700)	4,900 (3,700–7,800)	4,500 (3,000–6,600)	9:73	0.2921
Hemoglobin (g/dL)	9.7 (8.3-11.2)	9.6 (8.9-10.7)	9.7 (8.3-11.2)	9:73	0.9173
Platelets (*10^4^/μL)	1.3 (0.5-4.8)	1.4 (0.8-5.2)	1.1 (0.5-4.8)	9:73	0.6667
Albumin (mg/dL)	2.7 (2.4-3.1)	2.6 (2.4-3.1)	2.7 (2.4-3)	9:73	0.9763
Total bilirubin (mg/dL)	4.0 (2.1-9.2)	3.1 (2.4-8.3)	4 (2.1-9.6)	9:73	0.5678
Direct bilirubin (mg/dL)	1.5 (0.4-4.7)	1.3 (0.5-2.2)	1.6 (0.4-5.2)	9:72	0.5524
AST (U/L)	53 (37–77)	40 (24–44)	58 (38–78)	9:73	0.0680
ALT (U/L)	28 (22–50)	25 (20–31)	29 (23–52)	9:73	0.2379
ALP (U/L)	412 (289–691)	341 (276–433)	431 (289–693)	9:73	0.3937
UN (mg/dL)	16 (12–26)	24 (16–27)	15 (12–25)	9:73	0.1650
Creatinine (mg/mL)	0.8 (0.6-1.1)	1.2 (0.6-1.4)	0.8 (0.6-1.0)	9:73	0.3201
Estimated GFR (mL/min/1.73m^2^)	70 (49–89)	41 (37–75)	71 (51–89)	9:73	0.1380
Na (mEq/L)	137 (132–139)	134 (132–137)	137 (132–139)	9:73	0.6289
Hemoglobin A1c (%)	4.9 (4.4-5.4)	5.2 (4.6-6.1)	4.9 (4.3-5.4)	9:72	0.2656
C-reactive protein (mg/dL)	0.5 (0.1-1.2)	0.6 (0.6-1.3)	0.3 (0.1-1.1)	9:73	0.2352
Procalcitonin (ng/mL)	0.2 (0.1-0.3)	0.2 (0.2-0.3)	0.2 (0.1-0.3)	9:72	0.5328
Prothrombin time (second)	50 (39–61)	54 (46–58)	49 (39–61)	9:73	0.6562
Prothrombin time (INR)	1.5 (1.3-1.8)	1 (1–2)	2 (1–2)	9:73	0.5881
Activated partial thromboplastin time (second)	45 (40–57)	51 (40–78)	45 (41–56)	9:73	0.5044
NH3 (μg/dL)	76 (50–112)	109 (56–179)	74 (50–107)	9:73	0.1842
CEA (ng/mL)	4.0 (2.4-6.1)	6.2 (4.5-7.5)	3.9 (2.3-5.6)	8:71	0.0439**
CA 19-9 (U/mL)	30 (14–71)	9 (4–45)	30 (16–74)	8:71	0.1131
Alpha-fetoprotein (ng/mL)	6.3 (2.5-24.2)	5.5 (2.3-41.1)	6.7 (2.6-24.0)	9:73	0.6833
PIVKA-II (mAU/mL)	106 (37–429)	429 (27–1525)	101 (38–406)	9:73	0.2353
Total cholesterol (mg/dL)	118 (84–148)	105 (82–127)	118 (88–156)	4:38	0.5065
HDLC (mg/dL)	23 (7–39)	30 (25–35)	19 (7–39)	4:38	0.5341
LDLC (mg/dL)	50 (28–72)	51 (33–68)	50 (28–72)	4:38	1.0000
Triglyceride (mg/dL)	61 (42–100)	68 (60–77)	60 (42–100)	4:38	0.8137
IgM (mg/dL)	157 (89–208)	127 (118–137)	176 (88–229)	4:33	0.3920
IgA (mg/dL)	516 (357–734)	752 (554–892)	510 (357–726)	4:33	0.3655
IgG (mg/dL)	2117 (1684–2461)	2169 (1776–2284)	2114 (1684–2526)	8:69	0.8347
IgG at 1 week (mg/dL)	961 (750–1186)	1164 (953–1554)	926 (721–1185)	9:73	0.0365**
IgG at 2 weeks (mg/dL)	748 (583–1012)	885 (709–1504)	740 (576–960)	8:72	0.2023
IgG at 4 weeks (mg/dL)	870 (673–1112)	1107 (936–1270)	834 (670–1105)	6:69	0.1512
C3 (mg/dL)	63 (41–91)	65 (62–90)	62 (40–91)	8:58	0.4552
C3 at 1 week (mg/dL)	63 (49–75)	69 (53–77)	62 (48–75)	9:70	0.5218
C3 at 2 weeks (mg/dL)	85 (62–113)	56 (49–70)	88 (71–116)	8:67	0.0059**
C3 at 4 weeks (mg/dL)	113 (86–138)	89 (57–96)	120 (87–138)	5:62	0.0290**
C4 (mg/dL)	10 (7–14)	15 (11–16)	10 (7–13)	8:58	0.0883
C4 at 1 week (mg/dL)	11 (8–15)	12 (11–15)	11 (8–14)	9:70	0.3145
C4 at 2 weeks (mg/dL)	17 (11–22)	13 (9–15)	17 (12–22)	8:67	0.0531
C4 at 4 weeks (mg/dL)	22 (17–27)	17 (8–19)	23 (18–27)	5:62	0.0377**
Intravenous immunoglobulin	25 (30)	4 (44)	21 (29)	9:73	0.4445
Plasmapheresis	3 (4)	2 (22)	1 (1)	9:73	0.0306**
Fresh frozen plasma by day 90	71 (87)	9 (100)	62 (85)	9:73	0.6006
CMV infection by day 90	27 (33)	2 (22)	25 (34)	9:73	0.7106
Bacteremia by day 90	14 (17)	5 (56)	9 (12)	9:73	0.0061**
Infection by day 7	36 (44)	9 (100)	27 (37)	9:73	0.0003**
Infection by day 14	48 (59)	9 (100)	39 (53)	9:73	0.0089**
Infection by day 28	51 (62)	9 (100)	42 (58)	9:73	0.0118**
Infection by day 90	54 (66)	9 (100)	45 (62)	9:73	0.0245**
Early allograft dysfunction	26 (32)	7 (78)	19 (27)	9:73	0.0133**
Acute cellular rejection by day 14	3 (4)	1 (11)	2 (3)	9:73	0.2977

*Available cases (Non-survivors: Survivors), **<0.05 (Non-survivors vs. Survivors).

IQR, interquartile range; GW/SLV, graft volume/standard liver volume; MMF, mycophenolate mofetil; DD-reconstruction, duct-to-duct reconstruction; HCC, hepatocellular carcinoma; HBV, hepatitis B virus; HCV, hepatitis C virus; HLA, human leukocyte antigen; ICU, intensive care unit; AST, aspartate transaminase; ALT, alanine aminotransferase; ALP, alkaline phosphatase; UN, urea nitrogen; GFR, glomerular filtration rate; INR, international normalized ratio; CEA, carcinoembryonic antigen; CA 19-9, carbohydrate antigen; PIVKA-II, protein induced by vitamin K absence or antagonist-II; HDLC, high-density lipoprotein cholesterol; LDLC, low-density lipoprotein cholesterol; Ig, immunoglobulin; CMV, cytomegalovirus.

### Time-Dependent Changes in C3, C4, and IgG

**Figure 1:**
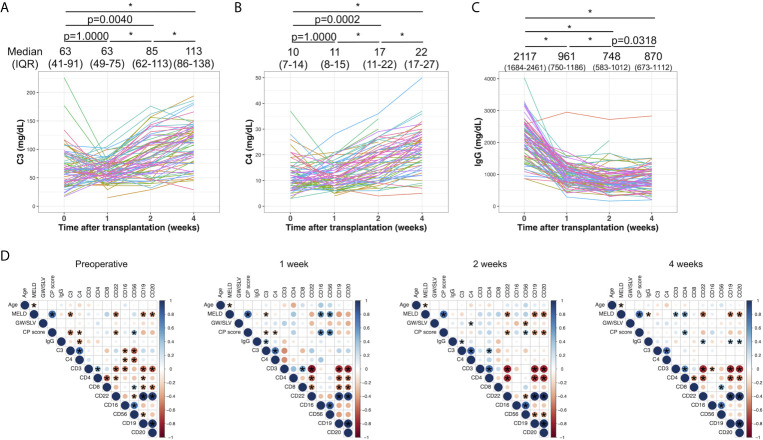
The time-dependent changes in serum C3 **(A)**, C4 **(B)**, and IgG **(C)**. Changes among 4 time points were evaluated by the Wilcoxon signed-rank test with Bonferroni correction. *p value<0.0001. The median values and interquartile ranges are shown at each of the time points. **(D)** Correlations between clinical and laboratory variables at the preoperative examination, and at 1, 2, and 4 weeks after LDLT. Blue and red indicate positive and negative correlations between two variables, respectively. An asterisk denotes a p value of <0.05 between two variables.

**Figures 1A–C** shows the time-dependent changes in serum C3, C4, and IgG. The median values and IQRs at each time point are shown in **Figures 1A–C**. The serum levels of C3 were comparable between the preoperative examination and at 1 week after LDLT (**Figure 1A**, p=1.0000). C3 continued to increase in a time-dependent manner until 4 weeks after LDLT (preoperative to 2 weeks: p=0.0040, 2 to 4 weeks: p<0.0001). The levels of C4 showed the same pattern of changes (**Figure 1B**, preoperative to 1 week: p=1.0000, preoperative to 2 weeks: p=0.0002, 2 to 4 weeks: p<0.0001). IgG decreased after LDLT and showed mild increases during the 4-week period (**Figure 1C**, preoperative to 2 weeks: p<0.0001, 2 to 4 weeks: p=0.0318), but did not return to the preoperative levels (preoperative to 4 weeks: p<0.0001). With the exception of 2 patients, all patients had IgG levels above the lower normal limit at the time of the preoperative examination. The changes in peripheral blood leukocyte populations determined by flow cytometry are shown in **Supplementary Figure 3**. We obtained the data of leukocyte populations from 37 patients at the preoperative examination, 19 patients at 1 week after LDLT, 32 patients at 2 weeks after LDLT, and 36 patients at 4 weeks after LDLT in this retrospective study.

### Correlations Between Clinical Parameters and Laboratory Results

**Figure 1D** shows the correlation between clinical parameters and laboratory results, including C3, C4, IgG, and the leukocyte populations at the preoperative examination, and at 1, 2, and 4 weeks after LDLT. C3 showed a mild negative correlation with the MELD score and Child–Pugh score (ρ=-0.40, p=0.0008 and ρ=-0.46, p<0.0001, respectively) at the preoperative examination. C3 and C4 were negatively correlated with CD16 and CD56 (C3 and CD16: ρ=-0.41, p=0.028, C3 and CD56: ρ=-0.55, p=0.0018, C4 and CD16: ρ=-0.38, p=0.043, C4 and CD56: ρ=-0.52, p=0.0042). This negative correlation disappeared at 1, 2, and 4 week(s) after LDLT. IgG had mild positive correlations with CD19, CD20, and CD22 at 4 weeks after LDLT (ρ=0.34, p=0.043; ρ=0.36, p=0.032; ρ=0.37, p=0.026, respectively).

### Characteristics of Survivors and Non-Survivors at Day 90

**Table 1** also shows the characteristics of non-survivors and survivors at day 90. Nine of 82 patients (11%) died within 90 days after LDLT. The causes of death included graft dysfunction, acute rejection, arterial thrombosis in the graft, infectious disease, and acute respiratory distress syndrome (**Supplementary Table 1**). The donor age of the non-survivors was older than that of the survivors (median [IQR]: 42 [39-54] years *vs.* 36 [28-44] years, p=0.0250). The preoperative laboratory data of both groups were comparable. Among the time-series measurements of C3, C4, and IgG, non-survivors had higher levels of IgG at 1 week after LDLT (1164 [953-1554] mg/dL *vs.* 926 [721-1185] mg/dL, p=0.0365) and lower levels of C3 at 2 and 4 weeks after LDLT (56 [49-70] mg/dL *vs.* 88 [71-116] mg/dL, p=0.0059; 89 [57-96] mg/dL *vs.* 120 [87-138] mg/dL, p=0.0290), and lower levels of C4 at 4 weeks after LDLT (17 [8-19] mg/dL *vs.* 23 [18-27] mg/dL, p=0.0377). Although fresh frozen plasma contains complement components, including C3 and C4 (29), survivors had used both plasmapheresis and fresh frozen plasma with comparable frequency to non-survivors. Non-survivors had more episodes of bacteremia and infection treated with antibiotics in comparison to survivors. Although CEA increased in non-survivors, there were no differences in hepatocellular carcinoma between survivors and non-survivors. Other malignancies were ruled out before transplantation.

### The Immunological Status, Characterized by C3, C4, IgG, and Leukocyte Populations, of Non-Survivors at 2 Weeks After LDLT

**Figure 2:**
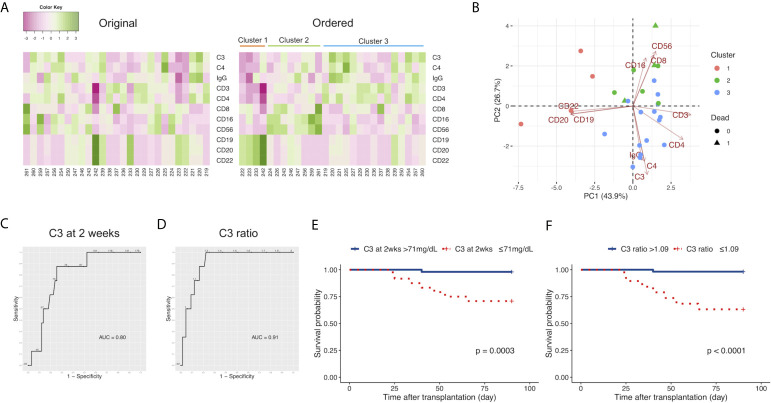
**(A)** Heat maps of the C3, C4, and IgG levels, and the leukocyte populations at 2 weeks after LDLT with the original order (left panel) and the k-means clustering order (right panel). The numbers below the heat maps show the case number of each patient. **(B)** The two-dimensional principal component analysis using C3, C4, and IgG, and the leukocyte populations at 2 weeks after LDLT. The colors indicate the cluster made by k-means clustering. Circles: survivors. Triangles: non-survivors. **(C)** The ROC curve for the serum levels of C3 at 2 weeks after LDLT to distinguish non-survivors from survivors at day 90. **(D)** The ROC curve for the ratio of C3 at 2 weeks after LDLT to C3 at 1 week after LDLT (C3 ratio) to distinguish non-survivors from survivors at day 90. **(E)** The Kaplan-Meier estimates for 90-day survival of patients with a C3 level at 2 weeks of ≤ 71 mg/dL and patients with a C3 level at 2 weeks of > 71 mg/dL. **(F)** The Kaplan-Meier estimates for 90-day survival of patients with a C3 ratio of >1.09 and patients with a C3 ratio of ≤1.09.

Because we observed that the levels of C3 at 2 weeks and 4 weeks after LDLT in non-survivors were lower than those in survivors, we sought to identify the comprehensive immunological status, characterized by C3 and other markers, including C4, IgG, and leukocyte populations of non-survivors at 2 weeks after LDLT. We performed k-means clustering and observed 3 clusters with relatively high levels of specific markers as follows: Cluster 1, characterized by markers of B cells (CD19, CD20, CD22); Cluster 2, characterized by markers of killer T cells (CD8), monocytes (CD16), and natural killer cells (CD56); and Cluster 3, characterized by markers of T cells (CD3), helper T cells (CD4), C3, C4, and IgG (**Figure 2A**). Furthermore, we performed a two-dimensional PCA using the C3, C4, IgG, and leukocyte populations at 2 weeks after LDLT (**Figure 2B**). The first principal component (PC 1) contained CD3 and CD4 on the positive side, and CD19, CD20, CD22 on the negative side. The second principal component (PC 2) contained CD8, CD16, CD56 on the positive side, and C3, C4, IgG on the negative side. Three clusters made by k-means clustering were plotted on the two axes of the principal components (**Figure 2B**). All 3 non-survivors were included in Cluster 2. We also performed the same analyses with the sub-population of patients excluding ABO-incompatible LDLT because all patients who underwent ABO-incompatible LDLT were treated with rituximab prior to LDLT, which critically affected the leukocyte populations. We found that all 2 non-survivors were included in Cluster 2, which was mainly characterized by CD8, CD16, and CD56. This result was same as that of the analysis of the overall patient population (**Supplementary Figure 4A**).

### The Cutoff Level of C3 at 2 Weeks to Distinguish Non-Survivors From Survivor

We sought to quantify how accurately C3 at 2 weeks could distinguish death within 90 days after LDLT using an ROC curve analysis. The cutoff value of C3 at 2 weeks was 71 mg/dL; this yielded sensitivity and specificity values of 87.5% and 75.0%, respectively (**Figure 2C**). The area under the ROC curve (AUC) for C3 at 2 weeks was 0.80. We focused on the time-dependent changes of C3 to improve the ability to distinguish non-survivors from survivors. We found a pattern in which survivors had relatively higher levels of C3 at 2 weeks in comparison to those at 1 week after LDLT. Therefore, we calculated the “ratio of C3 at 2 weeks/C3 at 1 week (C3 ratio)” and used this value to distinguish survivors from non-survivors. We determined the cutoff value of the C3 ratio for distinguishing non-survivors from survivors with an ROC curve analysis (**Figure 2D**). The cutoff value of the C3 ratio was 1.09. This yielded sensitivity and specificity values of 87.5% and 83.6%, respectively. The AUC for the C3 ratio was 0.91. C3 ratio improved the specificity in distinguishing survivors from non-survivors in comparison to C3 at 2 weeks.

### C3 and 90-Day Mortality

We divided the patients into two groups based on cutoff levels of C3 at 2 weeks after LDLT and the C3 ratio and compared their survival. Our cohort included no patients who died within 2 weeks. **Figure 2E** shows the survival rates at 90 days from LDLT of patients with serum C3 levels of ≤ 71 mg/dL and > 71 mg/dL at 2 weeks after LDLT. The C3 ≤ 71 mg/dL group showed a lower survival rate (p=0.0003). As for the C3 ratio, when 1.09 was used as the cutoff value of the C3 ratio (based on the results of the ROC curve analysis), the patients with a C3 ratio of ≤1.09 had a lower probability of survival (**Figure 2F**, p<0.0001). The odds ratio of C3 ratio ≤1.09 to 90-day mortality obtained from the weighted logistic regression was 13.07754 (95% confidence interval: 1.38513 to 123.47035, p=0.0279). The number of events per variable (EPV) was less than 10 (i.e., 4.0) in the analysis model, however, the estimated odds ratio was not substantially different from the estimated odds ratio from the univariate model (odds ratio: 12.68, 95% confidence interval: 1.39515 to 115.86768, p= 0.0272).

### Characteristics of Patients With and Without Infection Treated With Antibiotics by Day 7

We focused on the emergence of infection requiring antibiotic treatment by day 7 because all non-survivors had infections treated with antibiotics by day 7. **Table 2** shows the characteristics of patients with and without infection treated with antibiotics by day 7. Patients with infection treated with antibiotics by day 7 had more episodes of preoperative stay in the intensive care unit (ICU) (n [%]: 7 [19%] days *vs.* 2 [4%] days, p=0.0383). The laboratory data at the preoperative examination were comparable between the two groups.

**Table 2:** Clinical characteristics of patients with or without infection by day 7.

Clinical characteristics, median (IQR) or n (%)	With infection by day 7 (n = 36)	Without infection by day 7 (n = 46)	Number*	P value
Age (years)	59 (53–65)	60 (53–64)	36:46	0.6000
Sex (male)	18 (50)	27 (59)	36:46	0.5051
Hight (m)	1.63 (1.52-1.70)	1.61 (1.53-1.69)	36:46	0.7398
Body weight (kg)	66.5 (55.8-76.1)	59.5 (53.1-68.1)	36:46	0.2192
Body mass index (kg/m^2^)	24.4 (22.1-27.4)	23.4 (21.3-25.7)	36:46	0.2524
Child–Pugh score	11 (9–12)	11 (9–12)	36:46	0.8352
MELD score at transplantation	18 (14–27)	17 (13–22)	36:46	0.2537
GW/SLV	41 (33–48)	44 (34–54)	36:46	0.1956
Donor age (years)	39 (31–50)	37 (30–44)	36:46	0.4715
Donor sex (male)	21 (58)	22 (48)	36:46	0.3798
Cold ischemic time (minutes)	87 (61–103)	86 (59–96)	36:46	0.4950
Operative time (minutes)	790 (721–877)	746 (680–817)	36:46	0.1555
Blood loss (mL)	6,900 (4,300–13,000)	5,500 (3,500–8,400)	36:46	0.0953
Splenectomy	18 (50)	19 (41)	36:46	0.5051
Other immunosuppressants	31 (86)	30 (65)	36:46	0.0418**
DD-reconstruction	33 (92)	42 (91)	36:46	1.0000
HCC	12 (33)	18 (39)	36:46	0.6486
HBV positive	4 (11)	3 (7)	36:46	0.6935
HCV positive	13 (36)	13 (28)	36:46	0.4814
Alcoholic Cirrhosis	5 (14)	14 (30)	36:46	0.1135
Nonalcoholic fatty liver disease	2 (6)	4 (9)	36:46	0.6905
Preoperative ICU	7 (19)	2 (4)	36:46	0.0383**
Child-Pugh C	25 (69)	30 (65)	36:46	0.8137
ABO incompatible	12 (33)	9 (20)	36:46	0.2043
Preoperative bacteremia	3 (8)	2 (4)	36:46	0.6495
HLA mismatch	3 (2–3)	3 (2–4)	36:46	0.3150
White blood cells (/μL)	4700 (3500–6800)	4300 (2700–6700)	36:46	0.4546
Hemoglobin (g/dL)	9.7 (8.3-10.9)	9.7 (8.3-11.2)	36:46	0.9218
Platelets (*10^4^/μL)	1.1 (0.5-3.6)	1.5 (0.5-5.1)	36:46	0.2970
Albumin (mg/dL)	2.7 (2.5-3.2)	2.7 (2.4-3.0)	36:46	0.3708
Total bilirubin (mg/dL)	4.5 (2.0-10.1)	3.9 (2.1-8.1)	36:46	0.5942
Direct bilirubin (mg/dL)	1.8 (0.5-6.0)	1.2 (0.4-4.0)	36:45	0.3971
AST (U/L)	45 (37–71)	59 (39–77)	36:46	0.5251
ALT (U/L)	26 (22–37)	32 (23–60)	36:46	0.2638
ALP (U/L)	362 (283–487)	457 (293–848)	36:46	0.1785
UN (mg/dL)	16 (12–26)	17 (12–25)	36:46	0.7118
Creatinine (mg/mL)	0.8 (0.6-1.3)	0.8 (0.6-1.0)	36:46	0.4003
Estimated GFR (mL/min/1.73m^2^)	62 (41–90)	71 (56–88)	36:46	0.4056
Na (mEq/L)	137 (134–140)	136 (131–139)	36:46	0.0823
Hemoglobin A1c (%)	4.9 (4.4-5.3)	4.9 (4.3-5.5)	36:45	0.6859
C-reactive protein (mg/dL)	0.6 (0.2-1.5)	0.3 (0.1-1.0)	36:46	0.2427
Procalcitonin (ng/mL)	0.3 (0.1-0.4)	0.2 (0.1-0.2)	36:45	0.0598
Prothrombin time (second)	48 (39–56)	51 (40–65)	36:46	0.3356
Prothrombin time (INR)	1.5 (1.4-1.8)	1.5 (1.3-1.8)	36:46	0.3381
Activated partial thromboplastin time (second)	49 (40–62)	43 (40–55)	36:46	0.2372
NH3 (μg/dL)	88 (54–118)	72 (48–102)	36:46	0.4189
CEA (ng/mL)	4.3 (2.3-6.7)	3.9 (2.5-5.3)	35:44	0.4803
CA 19-9 (U/mL)	27 (7–76)	35 (17–68)	35:44	0.5406
Alpha-fetoprotein (ng/mL)	8.2 (2.3-32.6)	5.2 (2.7-15.0)	36:46	0.5655
PIVKA-II (mAU/mL)	46 (29–428)	114 (52–439)	36:46	0.3242
Total cholesterol (mg/dL)	124 (81–134)	106 (90–159)	15:27	0.5372
HDLC (mg/dL)	33 (7–45)	18 (8–38)	15:27	0.6270
LDLC (mg/dL)	49 (29–61)	50 (29–85)	15:27	0.4386
Triglyceride (mg/dL)	67 (42–103)	57 (43–91)	15:27	0.6365
IgM (mg/dL)	146 (118–189)	166 (88–229)	12:25	0.9741
IgA (mg/dL)	628 (304–769)	493 (369–726)	12:25	0.7952
IgG (mg/dL)	2054 (1561–2313)	2227 (1801–2573)	32:45	0.1790
IgG at 1 week (mg/dL)	1054 (803–1222)	897 (724–1147)	36:46	0.2335
IgG at 2 weeks (mg/dL)	799 (666–1134)	724 (541–906)	35:45	0.1625
IgG at 4 weeks (mg/dL)	937 (656–1112)	851 (691–1114)	31:44	0.7266
C3 (mg/dL)	64 (45–90)	62 (40–91)	28:38	0.7554
C3 at 1 week (mg/dL)	60 (47–70)	66 (53–78)	36:43	0.1042
C3 at 2 weeks (mg/dL)	75 (54–96)	100 (74–125)	34:41	0.0100**
C3 at 4 weeks (mg/dL)	97 (78–128)	122 (92–141)	27:40	0.0427**
C4 (mg/dL)	10 (9–15)	10 (7–13)	28:38	0.4823
C4 at 1 week (mg/dL)	11 (8–13)	11 (8–15)	36:43	0.5438
C4 at 2 weeks (mg/dL)	14 (10–20)	19 (12–23)	34:41	0.0578
C4 at 4 weeks (mg/dL)	21 (16–27)	24 (19–28)	27:40	0.1294
Intravenous immunoglobulin	16 (44)	9 (20)	36:46	0.0178**
Plasmapheresis	2 (6)	1 (2)	36:46	0.5793
Fresh frozen plasma by day 7	32 (89)	37 (80)	36:46	0.3705
CMV infection by day 90	14 (39)	13 (28)	36:46	0.3499
Bacteremia by day 90	13 (36)	1 (2)	36:46	0.0001**
Infection by day 14	36 (100)	12 (26)	36:46	<0.0001**
Infection by day 28	36 (100)	15 (33)	36:46	<0.0001**
Infection by day 90	36 (100)	18 (39)	36:46	<0.0001**
Early allograft dysfunction	20 (57)	6 (13)	36:46	<0.0001**
Acute cellular rejection by day 14	2 (6)	1 (2)	36:46	0.5793
Death by day 90	9 (25)	0 (0)	36:46	0.0003**

*Available cases (With infection by day 7: Without infection by day 7), **<0.05.

IQR, interquartile range; GW/SLV, graft volume/standard liver volume; MMF, mycophenolate mofetil; DD-reconstruction, duct-to-duct reconstruction; HCC, hepatocellular carcinoma; HBV, hepatitis B virus; HCV, hepatitis C virus; HLA, human leukocyte antigen; ICU, intensive care unit; AST, aspartate transaminase; ALT, alanine aminotransferase; ALP, alkaline phosphatase; UN, urea nitrogen; GFR, glomerular filtration rate; INR, international normalized ratio; CEA, carcinoembryonic antigen; CA 19-9, carbohydrate antigen; PIVKA-II, protein induced by vitamin K absence or antagonist-II; HDLC, high-density lipoprotein cholesterol; LDLC, low-density lipoprotein cholesterol; Ig, immunoglobulin; CMV, cytomegalovirus.

### The Preoperative Immunological Status Characterized by C3, C4, IgG, and the Leukocyte Populations of Patients With Infection by Day 7

**Figure 3:**
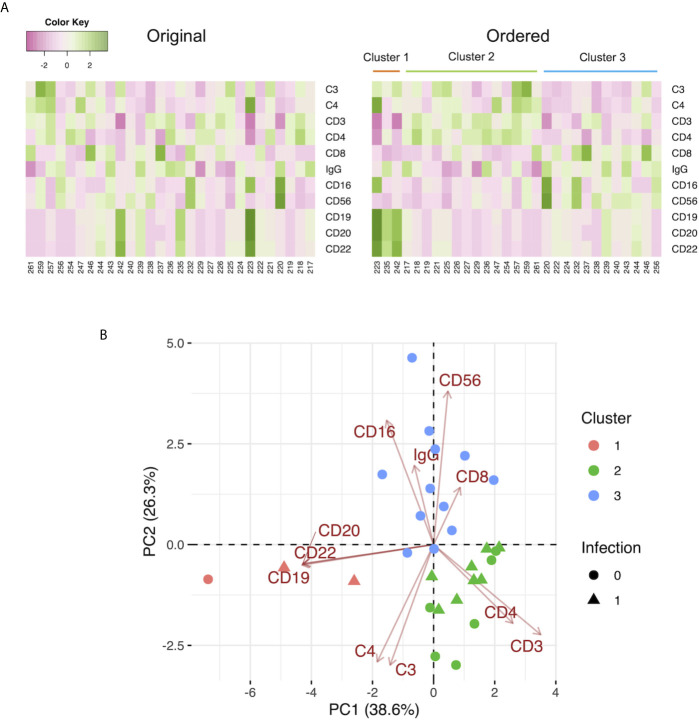
**(A)** Heat maps of the preoperative C3, C4, and IgG levels, and the leukocyte populations with the original order (left panel) and the k-means clustering order (right panel). The numbers below the heat maps show the case number of each patient. **(B)** The two-dimensional principal component analysis using the preoperative C3, C4, and IgG levels, and the leukocyte populations. The colors indicate the cluster made by k-means clustering. Circles: patients without infection by day 7. Triangles: patients with infection treated with antibiotics by day 7.

We performed k-means clustering using preoperative the C3, C4, IgG levels, and the leukocyte populations and observed 3 clusters as follows: Cluster 1, characterized by markers of B cells (CD19, CD20, CD22); Cluster 2, characterized by markers of T cells (CD3), helper T cells (CD4), C3, and C4; and Cluster 3, characterized by markers of killer T cells (CD8), monocytes (CD16), and natural killer cells (CD56), and IgG (**Figure 3A**). A two-dimensional PCA using preoperative C3, C4, IgG, and leukocyte populations showed the first principal component (PC 1) containing CD19, CD20, and CD22 on the negative side, and the second principal component (PC 2) containing CD8, CD16, CD56, and IgG on the positive side, and C3 and C4 on the negative side. Three clusters made by k-means clustering were plotted on the two axes of the principal components (**Figure 3B**). Patients with infection treated with antibiotics by day 7 were included in Clusters 1 and 2. In contrast, Cluster 3 had no patients with infection treated with antibiotics by day 7. After the exclusion of patients who underwent ABO-incompatible LDLT, the same analysis yielded similar results to those observed in the overall population (**Supplementary Figure 4B**).

### Characteristics of Patients With and Without CMV Infection by Day 90

**Supplementary Table 2** shows the characteristics of patients with and without CMV infection by day 90. Patients with CMV infection had lower levels of serum IgG at the preoperative examination (1837 [1575-2188] mg/dL *vs.* 2245 [1826-2575] mg/dL, p=0.0085), lower platelet counts (0.8 [0.4-2.5] *10^4^/μL *vs.* 1.8 [0.6-5.8] *10^4^/μL, p=0.0208), and more episodes of splenectomy (17 [63%] *vs.* 20 [36%], p=0.0333). The hemoglobin A1c (HbA1c) was value was slightly higher (5.2 [4.9-5.7] % *vs.* 4.8 [4.3-5.4] %, p=0.0252). The CMV serostatus of recipients could not be statistically analyzed because the number of patients was inadequate (CMV IgG positive: n=81, negative: n=1).

### The Preoperative Immunological Status, Characterized by C3, C4, IgG, and the Leukocyte Populations, of Patients With CMV Infection by Day 7

**Figure 4:**
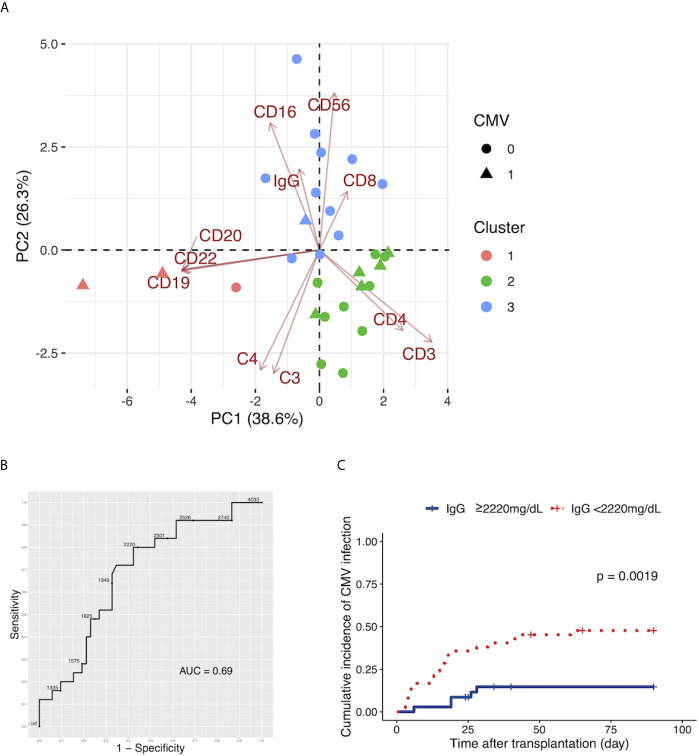
**(A)** The two-dimensional principal component analysis using the preoperative C3, C4, and IgG levels, and the leukocyte populations. The colors indicate the cluster made by k-means clustering. Circles: patients without cytomegalovirus (CMV) infection by day 90. Triangles: patients with CMV infection by day 90. **(B)** The ROC curve for the preoperative serum levels of IgG to distinguish patients with CMV infection by day 90 from patients without CMV infection by day 90. **(C)** The Kaplan-Meier estimates for the incidence of CMV infection by day 90 in patients with a preoperative IgG level of ≥2220 mg/dL and patients with a preoperative IgG level of <2220 mg/dL.

We evaluated the patients with CMV infection using clusters made by k-means clustering with the preoperative C3, C4, IgG, and leukocyte populations. Patients with CMV infection by day 7 were included in Clusters 1 and 2; only one patient with CMV infection was included in Cluster 3 (**Figure 4A**). After the exclusion of patients who underwent ABO-incompatible LDLT, the same analysis yielded similar results to those observed in the overall population (**Supplementary Figure 4C**).

### The Cutoff Level of Preoperative IgG for CMV Infection

We determined the cutoff values of preoperative serum IgG levels to distinguish patients with subsequent CMV infection from patients without subsequent CMV infection with an ROC curve analysis (**Figure 4B**). The cutoff value of preoperative IgG was 2,220 mg/dL. This yielded sensitivity and specificity values of 80.0% and 57.7%, respectively. The AUC for preoperative IgG was 0.69.

### Preoperative IgG and Incidence of CMV Infection

We divided patients into two groups, those with a preoperative IgG level of ≥2,220 mg/dL and patients with a preoperative IgG level of <2,220 mg/dL, and found a higher incidence of CMV infection in patients with preoperative IgG<2,220 mg/dL (**Figure 4C**, p=0.0019).

### The Multivariate Analysis of Factors Associated With CMV Infection by Day 90

To determine the variables that contributed to CMV infection, we performed a multivariate analysis using a Cox proportional hazards model. Among the variables, we used two variables with the smallest p values, because the number of incidents of CMV infection was 27. The results revealed that preoperative IgG had the strongest association with CMV infection (preoperative IgG; hazard ratio: 0.9988 (95% confidence interval: 0.9981-0.9996), p=0.00304. splenectomy; hazard ratio: 2.316 (95% confidence interval: 1.0305-5.2052), p=0.04209).

### Characteristics of Patients With and Without Bacteremia by Day 90

Our cohort included 14 patients with subsequent bacteremia by day 90 after LDLT. We evaluated the characteristics of patients with and without subsequent bacteremia (**Supplementary Table 3**). Significant differences were observed between the two groups in two factors: donor age (41 [39-52] years *vs.* 35 [28-45] years, p=0.0273) and episodes of preoperative stay at the ICU (4 [29%] *vs.* 5 [7%], p=0.0417).

## Discussion

We demonstrated that complement and IgG levels and leukocyte populations were associated with the outcomes of LDLT as follows: first, C3 and C4 increased in a time-dependent manner after LDLT, while IgG was dramatically reduced after LDLT. Second, low levels of serum C3 at 2 weeks were associated with 90-day mortality and the ratio of C3 at 2 weeks to C3 at 1 week improved the ability to distinguish survivors from non-survivors. Third, C3 and C4 showed an inverse relationship with the percentages of CD8, CD16, and CD56 in the PCA analysis. Fourth, patients with relatively higher percentages of CD8, CD16, and CD56 had fewer incidents of subsequent infection requiring treatment with antibiotics and fewer incidents of subsequent CMV infection.

We demonstrated a time-dependent increase in the serum levels of C3 and C4 after LDLT. Patients with chronic hepatic disease have low serum levels of complement (30). This may be due to impaired synthesis of complement and consumption of complement. Our patients also had lower levels of C3 and C4 in the preoperative examination. We observed an increase of C3 and C4 at 2 weeks after LDLT. We considered that two reasons explained the increase of C3 and C4 in the overall study population: the first is the incidence of infectious diseases; the second is the process of C3 and C4 normalization after LDLT. Infectious diseases cause an increase in complement components (C3, C4, C9, Factor B, C1 inhibitor, C4b-binding protein, Mannose-binding lectin) (31). In contrast, we found that patients with infection by day 7 had lower C3 levels in comparison to patients without infection by day 7 (**Table 2**). Thus, we concluded that infectious disease incidents were not the cause of the time-dependent increase in C3 and C4 and we assume that a time-dependent increase of C3 and C4 reflects the process of normalization of synthesis in the liver. In contrast, IgG drastically declined after LDLT, which is consistent with previous reports (32–34).

We observed that non-survivors at day 90 had lower levels of C3 at 2 weeks and demonstrated that C3 at 2 weeks and C3 ratio could distinguish survivors from non-survivors with the cutoff level as 71mg/dL and 1.09, respectively. The causes of hypocomplementemia consist of immune complex formation-related diseases (lupus erythematosus, glomerulonephritis, cryoglobulinemia, vasculitis as major causes) and others (35). Diseases and conditions in which factors other than immune complex formation are regarded as the cause of the hypocomplementemia include atheroembolism (cholesterol embolism), severe sepsis, hemolytic uremic syndrome (HUS), thrombotic thrombocytopenic purpura (TTP), thrombotic microangiopathy (TMA), and pancreatitis (35). Among these causes of hypocomplementemia, TMA (36), atheroembolism (37), and cryoglobulinemia (38) have been reported as complications in liver transplantation. In the present study, most patients who died by day 90 (non-survivors) had vascular events such as arterial thrombosis or infection (**Supplementary Table 1**). One of the non-survivors was clinically diagnosed as TMA at day 6, which possibly contributed to the hypocomplementemia. However, we could not identify the causes of hypocomplementemia in the other patients retrospectively because we had limited clinical information including only 2 cases of autopsy and 6 cases of post-mortem liver biopsies. The monitoring of serum C3 levels at 1 and 2 weeks after LDLT may distinguish subsequent mortality after transplantation and careful investigations of any complications causing hypocomplementemia may reduce the mortality rate.

We found that the ratio of C3 at 2 weeks to C3 at 1 week after LDLT was useful for distinguishing survivors from non-survivors. In healthy individuals, the half-life of C3, as determined using radioactive protein, is reported to be 64–81 hours (39). Based on this fact, C3 at 1 week after LDLT may reflect the synthesis by the donor liver. That the ratio of C3 at 2 weeks to C3 at 1 week after LDLT has better ability in distinguishing survivors from non-survivors may be because the ratio reflects both the baseline function of the donor liver after LDLT and the process of the normalization of the function of donor liver in the recipient. Although early graft dysfunction may cause low C3 ratio through the reduced production of complement, the results of our logistic regression analysis suggested that immunological mechanisms of hypocomplementemia other than early graft dysfunction and infection by day 7 underlie in non-survivors.

PCA using C3, C4, and IgG, and leukocyte populations demonstrated that C3 and C4 showed an inverse relationship with CD8, CD16, and CD56, both at the preoperative examination and at 2 weeks after LDLT. Especially, C3 and CD56 showed a negative correlation at the preoperative examination (ρ=-0.5542, p=0.0018) and at 2 weeks after LDLT (ρ=-0.2589, p=0.1922) (**Figure 1D**). CD56 is the major surface marker of natural killer (NK) cells and the ability of NK cells can be promptly activated by inflammatory cytokines, effector cytokines can be secreted, and infected or stressed host cells can be killed; thus, they are very early responders during infection (40). NK cell counts at 1 month after liver transplantation have been reported to predict the occurrence of opportunistic infection (41). Although we could not find any reports regarding NK cells and complement, it is important to confirm whether the contribution of C3 levels to 90-day mortality is the cause of death or whether it merely reflects the contributions of other factors, including NK cells.

Patients with relatively higher preoperative CD8, CD16, CD56, and IgG had fewer subsequent infectious diseases in comparison to other patients. We focused on the preoperative leukocyte populations, not the postoperative leukocyte populations, because 36 of 82 our patients had infectious diseases that required treatment with antibiotics by day 7 and several cases of CMV infection occurred by day 7. Although we suggested a cutoff value of IgG of 2,220 mg/dL to identify patients with a subsequent incident of CMV infection from those without CMV infection, it is important to distinguish the genuinely important factors among CD8, CD16, CD56, and IgG.

We observed that splenectomy prior to transplantation could be a risk factor for posttransplant CMV infection. Our results are compatible with the report of Neumann et al., who noted that simultaneous splenectomy with liver transplantation increased the incidence of opportunistic pneumonia, including CMV pneumonia (42).

The present study was associated with some limitations. First, we could not determine which of the three complement pathways was associated with liver transplantation because we only measured C3 and C4. C3 contributes relatively more in the alternative pathway and C4 is mainly associated with the classical and lectin pathways; however, distinguishing them clearly is difficult. Exhaustive measurement of complement components will resolve this matter in the future. Second, we did not have enough non-survivors to perform a multivariate analysis to evaluate the effect of each factor on mortality and to validate the accuracy of prediction considering the sampling variation because this unicenter study included a relatively limited number of patients. In addition, leukocyte population data was only present in 37 out of 82 patients. Furthermore, we did not have enough clinical information and detailed examinations to confirm the causes of hypocomplementemia including TMA, atheroembolism, and cryoglobulinemia because this research was a retrospective study. Our ongoing prospective study may resolve this matter. Third, we did not include monocyte subsets in leukocyte populations despite the suggestion that there is a role of monocyte subsets in liver diseases (43). Fourth, we have no evaluation of C3 polymorphism, although effects of C3 polymorphisms on outcomes of liver transplantation are reported (44, 45). C3 polymorphism may be associated with the serum levels of C3 in liver transplantation. Fifth, all but one of our patients were Japanese. Our results may not apply to patients in other regions.

In conclusion, we demonstrated a relationship between complement, IgG, and the leukocyte population and the usefulness of the C3 level at 2 weeks after LDLT in distinguishing survivors from non-survivors. Our results suggested the clinical importance of the immunological status, characterized by the complement and IgG levels and the peripheral leukocyte population in patients undergoing liver transplantation.

## Data Availability Statement

The datasets presented in this article are not readily available because we have no approval to make the datasets available to other researchers by the institutional review board. Requests to access the datasets should be directed to SE, (sueguchi@nagasaki-u.ac.jp).

## Ethics Statement 

The studies involving human participants were reviewed and approved by The Institutional Review Board of Nagasaki University Hospital. The patients/participants provided their written informed consent to participate in this study.

## Author Contributions 

SaF and MH conceptualized the project and collected the data with supervision by SE. SaF, MH, TH, AS, TA, HM, TT, and SE performed LDLT, including the perioperative management and maintained the records. MF, HH, and KY performed the flow cytometric analysis, and ShF and SM statistically analyzed the collected data. SE acquired the funding resource. SaF, ShF, SM, and HH wrote the original draft. All authors contributed to the article and approved the submitted version.

## Funding

This work was supported in part by a Grant-in-Aid for Research on HIV/AIDS from the Ministry of Health, Labour and Welfare of Japan.

## Conflict of Interest

The authors declare that the research was conducted in the absence of any commercial or financial relationships that could be construed as a potential conflict of interest.

## Supplementary Material

Supplementary Figure 1 The covariate balance between patients with a ratio of C3 at 2 weeks/C3 at 1 week after LDLT (C3 ratio) of ≤1.09 and those with C3 ratio of >1.09 in clinical factors associated with the serum C3 level. The differences between the two groups are displayed as the standardized mean difference (SMD).

Supplementary Figure 2 The distribution of the propensity scores. **(A)** The density of the propensity scores on the raw data. The red and green dots represent observations with the propensity score on the x-axis. **(B)** The same data as in Figure **(A)** were represented with a cumulative distribution function. **(C)** The density of the propensity score on the data after balancing using an inverse probability weight.

Supplementary Figure 3 The time-dependent changes in leukocyte populations. Changes among 4 time points were evaluated by the Wilcoxon signed-rank test. *p value<0.0001. The median values and interquartile ranges are shown at each time point.

Supplementary Figure 4
**(A)** The two-dimensional principal component analysis using the C3, C4, and IgG levels, and the leukocyte populations at 2 weeks after LDLT, after excluding patients who underwent ABO-incompatible LDLT. The colors indicate the cluster made by k-means clustering. Circles: survivors. Triangles: non-survivors. **(B)** The two-dimensional principal component analysis using the preoperative C3, C4, and IgG levels, and leukocyte populations, after excluding patients who underwent ABO-incompatible LDLT. The colors indicate the cluster made by k-means clustering. Circles: patients without infection by day 7. Triangle: patients with infection treated with antibiotics by day 7. **(C)** The two-dimensional principal component analysis using the preoperative C3, C4, and IgG levels, and the leukocyte populations, after excluding patients who underwent ABO-incompatible LDLT. The colors indicate the cluster made by k-means clustering. Circles: patients without cytomegalovirus (CMV) infection by day 90. Triangles: patients with CMV infection by day 90.
