# Corrigendum: The Contribution of Serum Complement Component 3 Levels to 90-Day Mortality in Living Donor Liver Transplantation

**DOI:** 10.3389/fimmu.2022.870480

**Published:** 2022-03-29

**Authors:** Saeko Fukui, Masaaki Hidaka, Shoichi Fukui, Shimpei Morimoto, Takanobu Hara, Akihiko Soyama, Tomohiko Adachi, Hajime Matsushima, Takayuki Tanaka, Mai Fuchigami, Hiroo Hasegawa, Katsunori Yanagihara, Susumu Eguchi

**Affiliations:** ^1^Department of Surgery, Nagasaki University Graduate School of Biomedical Sciences, Nagasaki, Japan; ^2^Program in Cellular and Molecular Medicine, Boston Children’s Hospital, Boston, MA, United States; ^3^Innovation Platform & Office for Precision Medicine, Nagasaki University Graduate School of Biomedical Sciences, Nagasaki, Japan; ^4^Department of Laboratory Medicine, Nagasaki University Graduate School of Biomedical Sciences, Nagasaki, Japan

**Keywords:** C3, C4, immunoglobulin G, posttransplant infection, leukocyte populations

In the published article, there was an error in affiliation 1. Instead of “Department of Surgery, Nagasaki University Graduate School of Biomedical Science, Nagasaki, Japan”, it should be “Department of Surgery, Nagasaki University Graduate School of Biomedical Sciences, Nagasaki, Japan”.

The authors apologize for this error and state that this does not change the scientific conclusions of the article in any way. The original article has been updated.

In the original article, there were several errors in the texts as follows.

1. A misspelled data unit was corrected from “md/dL” to “mg/dL”.

A correction has been made to the **Abstract**:

“Non-survivors had lower levels of C3 at 2 weeks in comparison to survivors (median [interquartile range]: 56 [49-70] mg/dL vs. 88 [71-116] mg/dL, p=0.0059)”.

2. One of the p values in the manuscript was written in a different format from the corresponding figure. Specifically, “p>0.9999” into “p=1.0000”.

A correction has been made to **RESULTS**, **Time-Dependent Changes in C3, C4, and IgG**:

“C3 continued to increase in a time-dependent manner until 4 weeks after LDLT (preoperative to 2 weeks: p=0.0040, 2 to 4 weeks: p<0.0001). The levels of C4 showed the same pattern of changes (**Figure 1B**, preoperative to 1 week: p=1.0000, preoperative to 2 weeks: p=0.0002, 2 to 4 weeks: p<0.0001)”.

3. The description of p values has been corrected from “p=0.02793” to “p=0.0279” for the sake of consistency throughout the article.

A correction has been made to **RESULTS, C3 and 90-Day Mortality**:

“The odds ratio of C3 ratio ≤1.09 to 90-day mortality obtained from the weighted logistic regression was 13.07754 (95% confidence interval: 1.38513 to 123.47035, p=0.0279). The number of events per variable (EPV) was less than 10 (i.e., 4.0) in the analysis model, however, the estimated odds ratio was not substantially different from the estimated odds ratio from the univariate model (odds ratio: 12.68, 95% confidence interval: 1.39515 to 115.86768, p=0.0272)”.

4. A paragraph to state our conclusion was removed by mistake during the revision process.

The following sentences have been inserted as a new paragraph in **DISCUSSION**, after paragraph 8:

“In conclusion, we demonstrated a relationship between complement, IgG, and the leukocyte population and the usefulness of the C3 level at 2 weeks after LDLT in distinguishing survivors from non-survivors. Our results suggested the clinical importance of the immunological status, characterized by the complement and IgG levels and the peripheral leukocyte population in patients undergoing liver transplantation”.

The authors apologize for this error and state that this does not change the scientific conclusions of the article in any way. The original article has been updated.

## Publisher’s Note

All claims expressed in this article are solely those of the authors and do not necessarily represent those of their affiliated organizations, or those of the publisher, the editors and the reviewers. Any product that may be evaluated in this article, or claim that may be made by its manufacturer, is not guaranteed or endorsed by the publisher.

