# Supplementary figures and images for: The Contribution of Serum Complement Component 3 Levels to 90-Day Mortality in Living Donor Liver Transplantation
Source: Front Immunol. 2021 Jul 19;12:652677. doi: 10.3389/fimmu.2021.652677 (PMC8326795; doi:10.3389/fimmu.2021.652677)

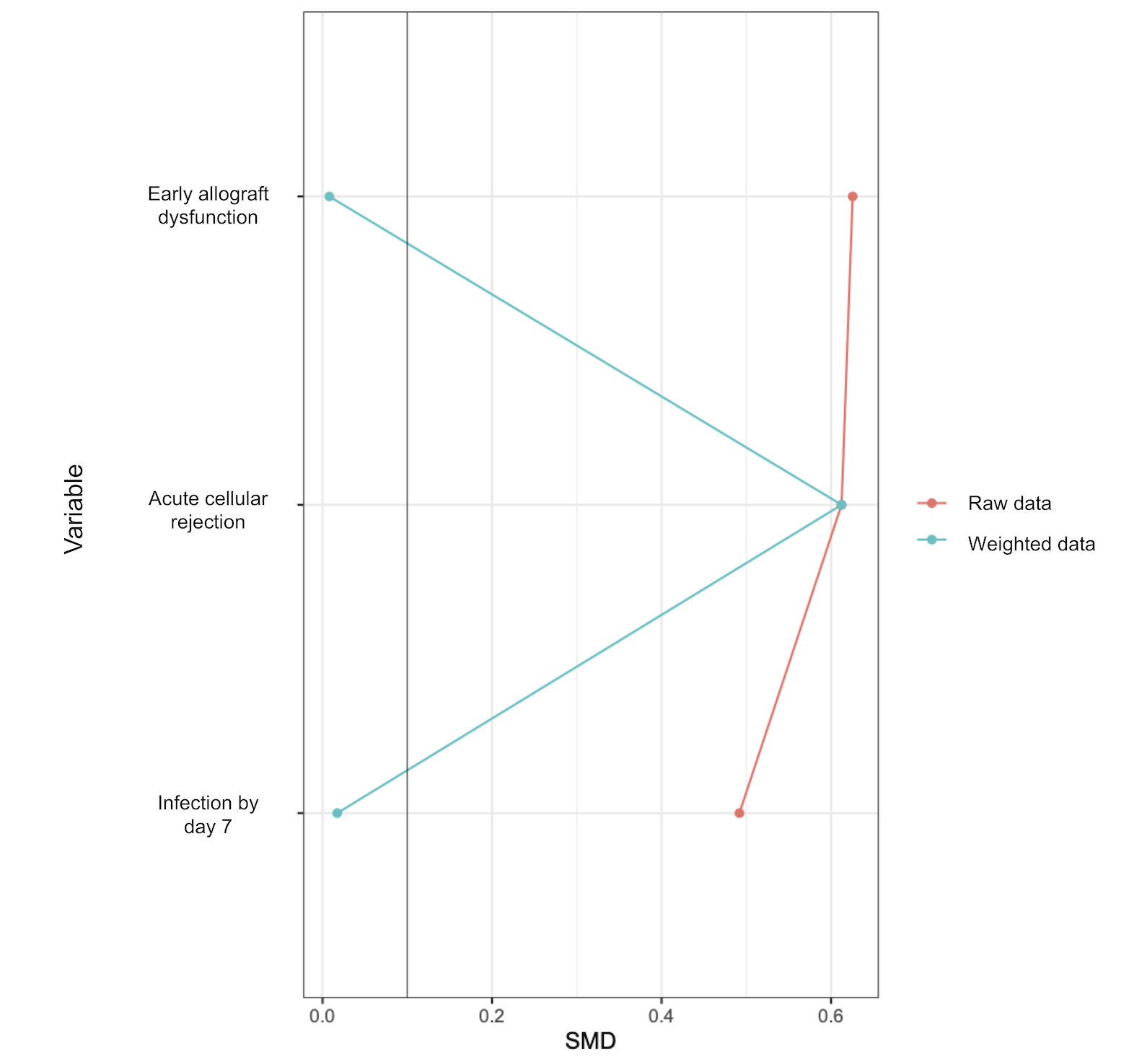

Supplement: Supplementary Figure 1 — The covariate balance between patients with a ratio of C3 at 2 weeks/C3 at 1 week after LDLT (C3 ratio) of ≤1.09 and those with C3 ratio of >1.09 in clinical factors associated with the serum C3 level. The differences between the two groups are displayed as the standardized mean difference (SMD). [file Image_1.tiff]

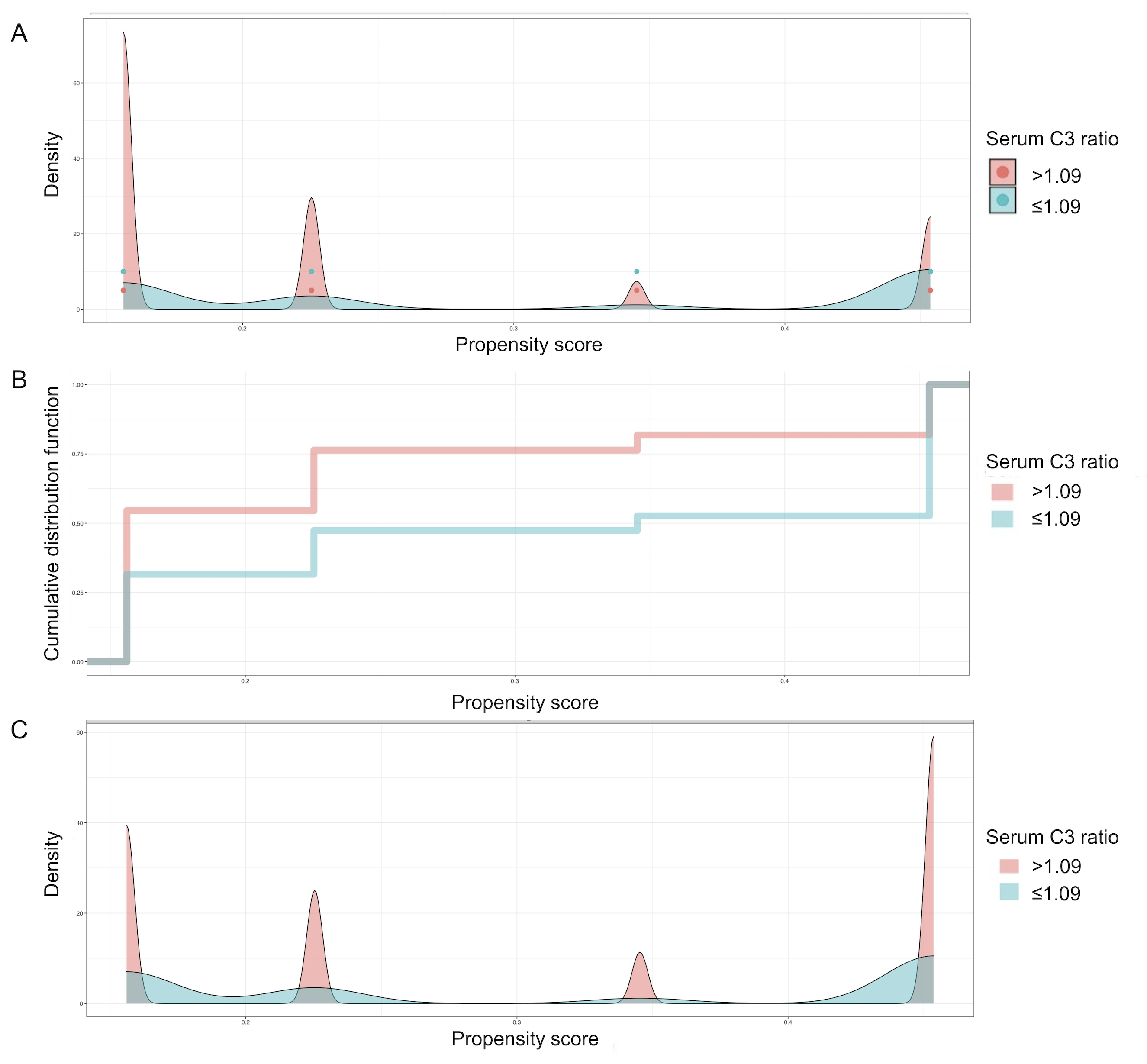

Supplement: Supplementary Figure 2 — The distribution of the propensity scores. (A) The density of the propensity scores on the raw data. The red and green dots represent observations with the propensity score on the x-axis. (B) The same data as in Figure (A) were represented with a cumulative distribution function. (C) The density of the propensity score on the data after balancing using an inverse probability weight. [file Image_2.tiff]

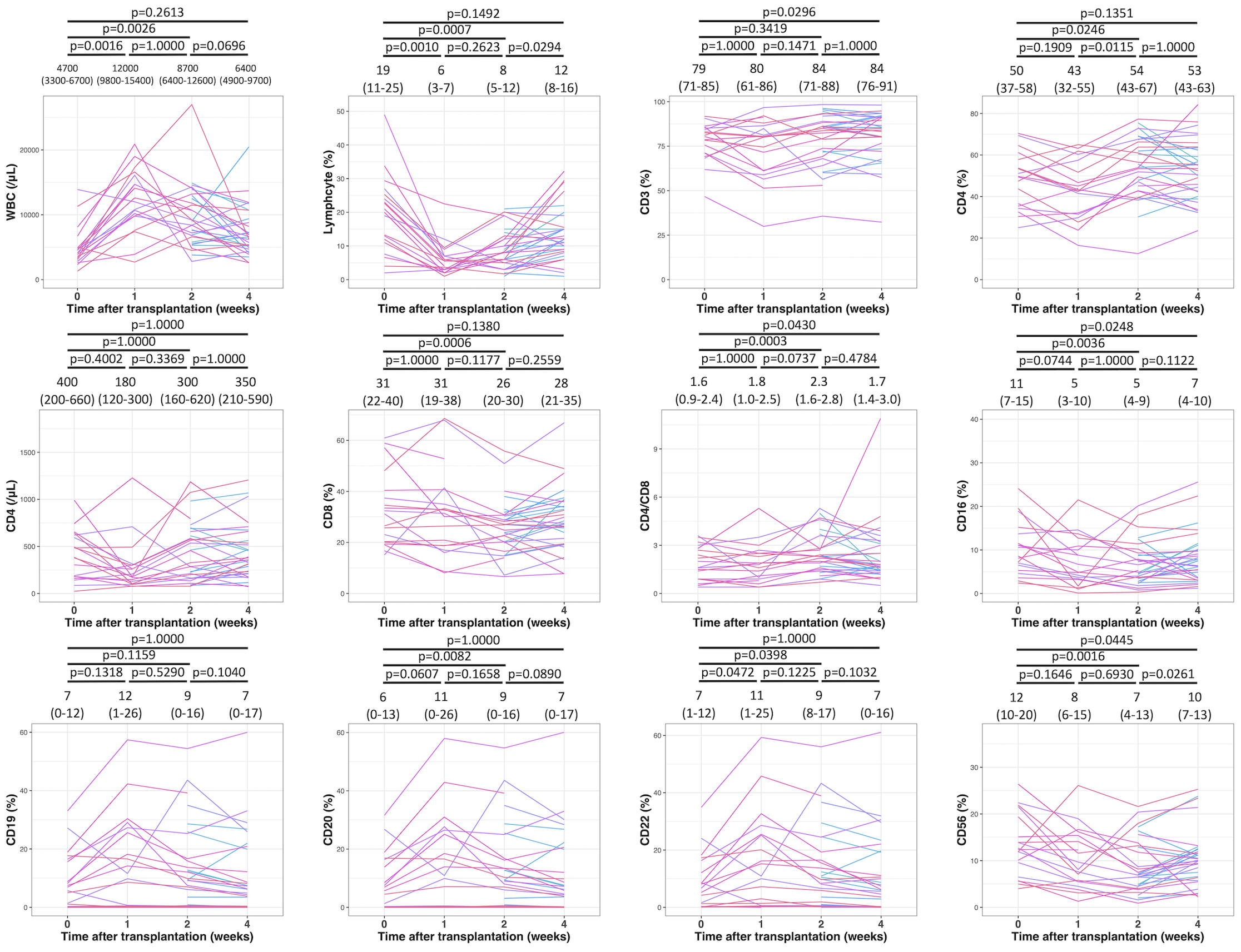

Supplement: Supplementary Figure 3 — The time-dependent changes in leukocyte populations. Changes among 4 time points were evaluated by the Wilcoxon signed-rank test. *p value<0.0001. The median values and interquartile ranges are shown at each time point. [file Image_3.tiff]

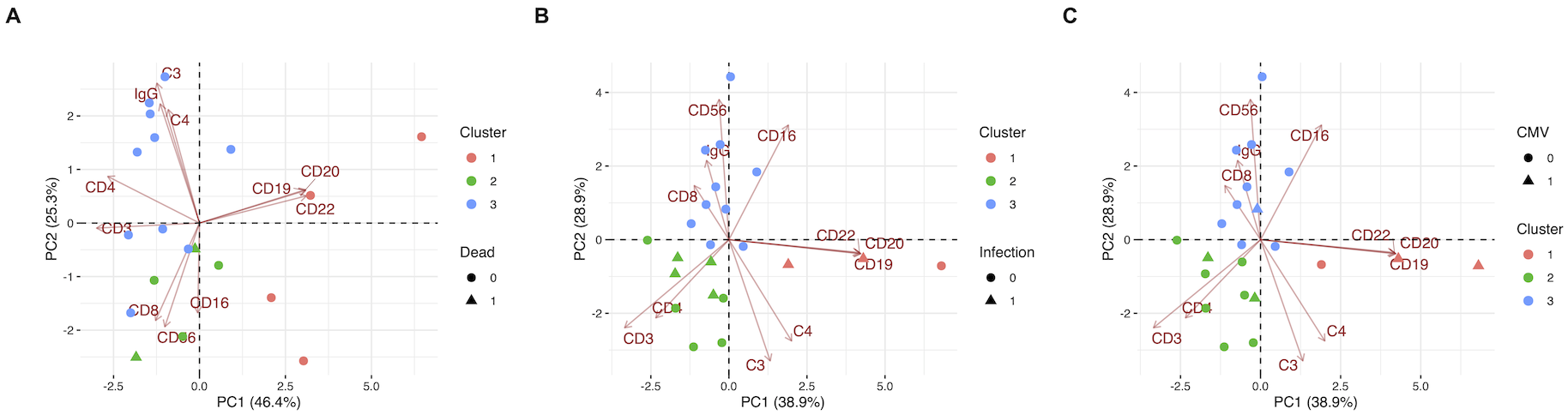

Supplement: Supplementary Figure 4 — (A) The two-dimensional principal component analysis using the C3, C4, and IgG levels, and the leukocyte populations at 2 weeks after LDLT, after excluding patients who underwent ABO-incompatible LDLT. The colors indicate the cluster made by k-means clustering. Circles: survivors. Triangles: non-survivors. (B) The two-dimensional principal component analysis using the preoperative C3, C4, and IgG levels, and leukocyte populations, after excluding patients who underwent ABO-incompatible LDLT. The colors indicate the cluster made by k-means clustering. Circles: patients without infection by day 7. Triangle: patients with infection treated with antibiotics by day 7. (C) The two-dimensional principal component analysis using the preoperative C3, C4, and IgG levels, and the leukocyte populations, after excluding patients who underwent ABO-incompatible LDLT. The colors indicate the cluster made by k-means clustering. Circles: patients without cytomegalovirus (CMV) infection by day 90. Triangles: patients with CMV infection by day 90. [file Image_4.tiff]
