# Supplementary material for: The Contribution of Serum Complement Component 3 Levels to 90-Day Mortality in Living Donor Liver Transplantation
Source: Front Immunol. 2021 Jul 19;12:652677. doi: 10.3389/fimmu.2021.652677 (PMC8326795; doi:10.3389/fimmu.2021.652677)
Supplement: Supplementary file 5 [file Table_1.docx]

Supplementary Table 1. Causes of death

| Case No. | Cause of death | Post-operative events  by day 14 | Pathological findings | C3 at day 14  (mg/dL) |
| --- | --- | --- | --- | --- |
| 1 | Graft dysfunction | Arterial thrombosis (day 3)  Infectious disease (day 7)  Bowel perforation (day13) | NA | 36 |
| 2 | Graft dysfunction | Portal vein stenosis (day 7)  Infectious disease (day 9) | NA | 49 |
| 3 | Infectious disease | Steroid pulse therapy for acute cellular rejection (day 8)  Infectious disease (day 12) | NA | 52 |
| 4 | Graft dysfunction | Duodenal bleeding (day 1)  Infectious disease (day 5) | Necrotic change. Organized thrombus in hepatic central vein at necrotic site, compatible.  (Post-mortem liver biopsy) | NA |
| 5 | Graft dysfunction | Thrombotic microangiopathy (day 6)  Infectious disease (day 8) | Granulation with foreign body reaction, necrosis, hemorrhage, and thrombi at hepatic hilum.  (Autopsy) | 60 |
| 6 | Acute respiratory distress syndrome | Infectious disease (day 2)  Inferior vena cava thrombosis (day 14) | Cholestasis and hepatocellular degeneration.  (Post-mortem liver biopsy) | 95 |
| 7 | Graft dysfunction, Infectious disease | Artery intimal dissection (intraoperative)  Infectious disease (day 3) | Zonal necrosis and cholestasis.  (Post-mortem liver biopsy) | 69 |
| 8 | Infectious disease | Infectious disease (day 5)  Embolization of collateral vessels (day 9) | Circulatory disturbance and severe bile stasis.  (Autopsy) | 71 |
| 9 | Infectious disease | Infectious disease (day 2) | Cholestasis. Circulatory disturbance, compatible.  (Post-mortem liver biopsy) | 48 |
