# Supplementary material for: The Contribution of Serum Complement Component 3 Levels to 90-Day Mortality in Living Donor Liver Transplantation
Source: Front Immunol. 2021 Jul 19;12:652677. doi: 10.3389/fimmu.2021.652677 (PMC8326795; doi:10.3389/fimmu.2021.652677)
Supplement: Supplementary file 6 [file Table_2.docx]

Supplementary Table 2. Clinical characteristics of patients with or without CMV by day 90.

| **Clinical characteristics,** **median (IQR) or n (%)** | **﻿With CMV (n=27)** | **﻿Without CMV (n=55)** | **Number*** | **P value** |
| --- | --- | --- | --- | --- |
| Age (years) | 59 (57-65) | 59 (52-63) | 27:55 | 0.2473 |
| Sex (male) | 14 (52) | 31 (56) | 27:55 | 0.8142 |
| Hight (m) | 1.61 (1.53-1.66) | 1.62 (1.53-1.70) | 27:55 | 0.2398 |
| Body weight (kg) | 62.6 (57.0-72.5) | 61.5 (52.7-73.0) | 27:55 | 0.9882 |
| Body mass index (kg/m^2^) | 24.1 (22.0-27.3) | 23.5 (21.2-26.4) | 27:55 | 0.4072 |
| Child–Pugh score | 11 (9-13) | 11 (9-12) | 27:55 | 0.3609 |
| MELD score at transplantation | 18 (12-25) | 16 (13-23) | 27:55 | 0.9370 |
| GW/SLV | 41 (33-50) | 42 (34-52) | 27:55 | 0.4016 |
| Donor age (years) | 36 (31-48) | 39 (30-45) | 27:55 | 0.9292 |
| Donor sex (male) | 11 (41) | 32 (58) | 27:55 | 0.1628 |
| Cold ischemic time (minutes) | 83 (61-96) | 86 (61-98) | 27:55 | 0.9292 |
| Operative time (minutes) | 784 (673-882) | 749 (688-854) | 27:55 | 0.9449 |
| Blood loss (mL) | 7,000 (3,300-9,800) | 5,500 (3,800-9,700) | 27:55 | 0.9607 |
| Splenectomy | 17 (63) | 20 (36) | 27:55 | 0.0333** |
| Other immunosuppressants | 18 (67) | 43 (78) | 27:55 | 0.2952 |
| DD-reconstruction | 27 (100) | 48 (87) | 27:55 | 0.0894 |
| HCC | 10 (37) | 20 (36) | 27:55 | 1.0000 |
| HBV positive | 4 (15) | 3 (5) | 27:55 | 0.2105 |
| HCV positive | 6 (22) | 20 (36) | 27:55 | 0.2188 |
| ﻿Alcoholic Cirrhosis | 6 (22) | 13 (24) | 27:55 | 1.0000 |
| Nonalcoholic fatty liver disease | 3 (11) | 3 (5) | 27:55 | 0.3899 |
| Preoperative ICU | 5 (19) | 4 (7) | 27:55 | 0.1471 |
| Child-Pugh C | 17 (63) | 38 (69) | 27:55 | 0.6225 |
| ABO incompatible | 9 (33) | 12 (22) | 27:55 | 0.2902 |
| Preoperative bacteremia | 3 (11) | 2 (4) | 27:55 | 0.3250 |
| HLA mismatch | 3 (2-4) | 3 (2-3) | 27:55 | 0.8905 |
| White blood cells (/μL) | 4400 (2700-8000) | 4600 (3300-6100) | 27:55 | 0.7709 |
| Hemoglobin (g/dL) | 9.8 (8.9-11.8) | 9.6 (8.3-10.9) | 27:55 | 0.5471 |
| Platelets (*10^4^/μL) | 0.8 (0.4-2.5) | 1.8 (0.6-5.8) | 27:55 | 0.0208** |
| Albumin (mg/dL) | 2.7 (2.5-3.0) | 2.6 (2.4-3.2) | 27:55 | 0.4580 |
| Total bilirubin (mg/dL) | 4.5 (2.0-10.5) | 3.8 (2.2-8.2) | 27:55 | 0.6498 |
| Direct bilirubin (mg/dL) | 1.8 (0.4-5.9) | 1.4 (0.5-4.1) | 26:55 | 0.6450 |
| AST (U/L) | 44 (37-74) | 58 (38-77) | 27:55 | 0.6044 |
| ALT (U/L) | 26 (20-47) | 29 (23-54) | 27:55 | 0.5803 |
| ALP (U/L) | 339 (275-472) | 433 (305-815) | 27:55 | 0.0790 |
| UN (mg/dL) | 16 (13-30) | 16 (12-25) | 27:55 | 0.6179 |
| Creatinine (mg/mL) | 0.8 (0.6-1.0) | 0.8 (0.6-1.2) | 27:55 | 0.4443 |
| Estimated GFR (mL/min/1.73m^2^) | 71 (50-91) | 68 (47-87) | 27:55 | 0.5738 |
| Na (mEq/L) | 137 (132-141) | 136 (132-139) | 27:55 | 0.3814 |
| Hemoglobin A1c (%) | 5.2 (4.9-5.7) | 4.8 (4.3-5.4) | 26:55 | 0.0252** |
| C-reactive protein (mg/dL) | 0.6 (0.1-1.1) | 0.5 (0.1-1.1) | 27:55 | 0.8979 |
| Procalcitonin (ng/mL) | 0.2 (0.1-0.4) | 0.2 (0.1-0.3) | 26:55 | 0.5043 |
| Prothrombin time (second) | 44 (39-54) | 54 (42-65) | 27:55 | 0.0982 |
| Prothrombin time (INR) | 1.6 (1.4-1.9) | 1.4 (1.3-1.7) | 27:55 | 0.1582 |
| Activated partial thromboplastin time (second) | 50 (42-67) | 44 (38-55) | 27:55 | 0.2119 |
| NH3 (μg/dL) | 82 (44-128) | 72 (51-101) | 27:55 | 0.6322 |
| CEA (ng/mL) | 4.2 (2.7-6.4) | 3.9 (2.3-5.6) | 25:54 | 0.5981 |
| CA 19-9 (U/mL) | 38.1 (16.1-78.8) | 25.7 (10.175-59.025) | 25:54 | 0.2021 |
| Alpha-fetoprotein (ng/mL) | 8 (4-27) | 5 (2-18) | 27:55 | 0.2564 |
| PIVKA-II (mAU/mL) | 50 (28-384) | 127 (41-492) | 27:55 | 0.1336 |
| Total cholesterol (mg/dL) | 113 (74-130) | 124 (86-154) | 11:31 | 0.4398 |
| HDLC (mg/dL) | 19 (5-40) | 27 (10-39) | 11:31 | 0.6367 |
| LDLC (mg/dL) | 55 (31-63) | 49 (29-81) | 11:31 | 0.7857 |
| Triglyceride (mg/dL) | 70 (39-95) | 58 (44-99) | 11:31 | 0.7968 |
| IgM (mg/dL) | 128 (79-148) | 176 (106-234) | 9:28 | 0.0927 |
| IgA (mg/dL) | 403 (265-658) | 581 (418-762) | 9:28 | 0.1902 |
| IgG (mg/dL) | 1837 (1575-2188) | 2245 (1826-2575) | 25:52 | 0.0085** |
| IgG at 1 week (mg/dL) | 929 (727-1206) | 968 (767-1186) | 27:55 | 0.5440 |
| IgG at 2 weeks (mg/dL) | 715 (598-935) | 790 (580-1054) | 26:54 | 0.4913 |
| IgG at 4 weeks (mg/dL) | 904 (616-1096) | 869 (695-1137) | 25:50 | 0.5037 |
| C3 (mg/dL) | 60 (40-78) | 65 (43-92) | 22:44 | 0.4340 |
| C3 at 1 week (mg/dL) | 66 (51-75) | 62 (49-75) | 27:52 | 0.8281 |
| C3 at 2 weeks (mg/dL) | 92 (62-116) | 80 (69-112) | 26:49 | 0.6124 |
| C3 at 4 weeks (mg/dL) | 120 (78-138) | 113 (88-134) | 23:44 | 0.9316 |
| C4 (mg/dL) | 10 (8-12) | 11 (7-15) | 22:44 | 0.3607 |
| C4 at 1 week (mg/dL) | 11 (8-14) | 11 (9-15) | 27:52 | 0.7717 |
| C4 at 2 weeks (mg/dL) | 18 (10-23) | 17 (12-21) | 26:49 | 0.7933 |
| C4 at 4 weeks (mg/dL) | 20 (16-28) | 23 (19-26) | 23:44 | 0.5129 |
| Donor CMV IgG positive | 27 (100) | 47 (85) | 27:55 | 0.0890 |
| Intravenous immunoglobulin | 10 (37) | 15 (27) | 27:55 | 0.4461 |
| Plasmapheresis | 1 (4) | 2 (4) | 27:55 | 1.0000 |
| Fresh frozen plasma by day 90 | 25 (93) | 46 (84) | 27:55 | 0.3242 |
| Bacteremia by day 90 | 4 (15) | 10 (18) | 27:55 | 1.0000 |
| Infection by day 7 | 14 (52) | 22 (40) | 27:55 | 0.3499 |
| Infection by day 14 | 19 (70) | 29 (53) | 27:55 | 0.1564 |
| Infection by day 28 | 20 (74) | 31 (56) | 27:55 | 0.1496 |
| Infection by day 90 | 21 (78) | 33 (60) | 27:55 | 0.1403 |
| Early allograft dysfunction | 10 (37) | 16 (30) | 27:55 | 0.6547 |
| Acute cellular rejection by day 14 | 1 (4) | 2 (4) | 27:55 | 1.0000 |
| Death by day 90 | 2 (7) | 7 (13) | 27:55 | 0.7106 |

*Available cases (With CMV : Without CMV), **<0.05.

**Abbreviations:** IQR, interquartile range, GW/SLV, graft volume/standard liver volume; MMF, mycophenolate mofetil; DD-reconstruction, duct-to-duct reconstruction; HCC, hepatocellular carcinoma; HBV, hepatitis B virus; HCV, hepatitis C virus; HLA, human leukocyte antigen; ICU, intensive care unit; AST, aspartate transaminase; ALT, alanine aminotransferase; ALP, alkaline phosphatase; UN, urea nitrogen; GFR, glomerular filtration rate; INR, international normalized ratio; CEA, carcinoembryonic antigen; CA 19-9, carbohydrate antigen; PIVKA-II, protein induced by vitamin K absence or antagonist-II; HDLC, high-density lipoprotein cholesterol; LDLC, low-density lipoprotein cholesterol; Ig, immunoglobulin; CMV, cytomegalovirus.
