# Supplementary material for: The Contribution of Serum Complement Component 3 Levels to 90-Day Mortality in Living Donor Liver Transplantation
Source: Front Immunol. 2021 Jul 19;12:652677. doi: 10.3389/fimmu.2021.652677 (PMC8326795; doi:10.3389/fimmu.2021.652677)
Supplement: Supplementary file 7 [file Table_3.docx]

Supplementary Table 3. Clinical characteristics of patients with or without bacteremia by day 90.

| **Clinical characteristics,** **median (IQR) or n (%)** | **﻿With bacteremia (n=14)** | **﻿Without bacteremia (n=68)** | **Number*** | **P value** |
| --- | --- | --- | --- | --- |
| Age (years) | 61 (54-65) | 59 (53-63) | 14:68 | 0.5088 |
| Sex (male) | 6 (43) | 39 (57) | 14:68 | 0.3839 |
| Hight (m) | 1.59 (1.52-1.70) | 1.62 (1.53-1.68) | 14:68 | 0.8823 |
| Body weight (kg) | 63.5 (52.6-76.0) | 61.6 (56.1-71.5) | 14:68 | 0.9951 |
| Body mass index (kg/m^2^) | 22.9 (21.6-25.8) | 23.7 (21.5-26.8) | 14:68 | 0.8873 |
| Child–Pugh score | 11 (9-12) | 11 (9-12) | 14:68 | 0.9354 |
| MELD score at transplantation | 18 (14-27) | 17 (13-22) | 14:68 | 0.4896 |
| GW/SLV | 42 (34-47) | 42 (33-52) | 14:68 | 0.7347 |
| Donor age (years) | 41 (39-52) | 35 (28-45) | 14:68 | 0.0273** |
| Donor sex (male) | 7 (50) | 36 (53) | 14:68 | 1.0000 |
| Cold ischemic time (minutes) | 87 (62-90) | 86 (60-98) | 14:68 | 1.0000 |
| Operative time (minutes) | 788 (697-875) | 751 (686-853) | 14:68 | 0.7300 |
| Blood loss (mL) | 5,500 (3,400-11,000) | 5,800 (3,700-9,100) | 14:68 | 0.9264 |
| Splenectomy | 5 (36) | 32 (47) | 14:68 | 0.5596 |
| Other immunosuppressants | 11 (79) | 50 (74) | 14:68 | 1.0000 |
| DD-reconstruction | 13 (93) | 62 (91) | 14:68 | 1.0000 |
| HCC | 4 (29) | 26 (38) | 14:68 | 0.5583 |
| HBV positive | 2 (14) | 5 (7) | 14:68 | 0.3419 |
| HCV positive | 3 (21) | 23 (34) | 14:68 | 0.5312 |
| ﻿Alcoholic Cirrhosis | 2 (14) | 17 (25) | 14:68 | 0.5033 |
| Nonalcoholic fatty liver disease | 2 (14) | 4 (6) | 14:68 | 0.2706 |
| Preoperative ICU | 4 (29) | 5 (7) | 14:68 | 0.0417** |
| Child-Pugh C | 10 (71) | 45 (66) | 14:68 | 1.0000 |
| ABO incompatible | 4 (29) | 17 (25) | 14:68 | 0.7471 |
| Preoperative bacteremia | 1 (7) | 4 (6) | 14:68 | 1.0000 |
| HLA mismatch | 3 (2-3) | 3 (2-3) | 14:68 | 0.8785 |
| White blood cells (/μL) | 4800 (3400-7600) | 4400 (3000-6600) | 14:68 | 0.3151 |
| Hemoglobin (g/dL) | 9.8 (8.5-11.8) | 9.7 (8.3-10.9) | 14:68 | 0.4301 |
| Platelets (*10^4^/μL) | 1.2 (0.5-1.9) | 1.4 (0.5-4.9) | 14:68 | 0.6304 |
| Albumin (mg/dL) | 3.0 (2.4-3.5) | 2.7 (2.4-3.0) | 14:68 | 0.3079 |
| Total bilirubin (mg/dL) | 7.6 (2.6-9.9) | 3.8 (2.1-8.3) | 14:68 | 0.2109 |
| Direct bilirubin (mg/dL) | 3.0 (0.7-6.6) | 1.3 (0.4-4.5) | 14:67 | 0.2022 |
| AST (U/L) | 62 (44-107) | 50.0 (36-73) | 14:68 | 0.1618 |
| ALT (U/L) | 30 (24-54) | 27 (22-49) | 14:68 | 0.5663 |
| ALP (U/L) | 387 (310-492) | 430 (288-695) | 14:68 | 0.7393 |
| UN (mg/dL) | 17 (13-23) | 16 (12-26) | 14:68 | 0.7672 |
| Creatinine (mg/mL) | 0.9 (0.7-1.2) | 0.8 (0.6-1.1) | 14:68 | 0.4939 |
| Estimated GFR (mL/min/1.73m^2^) | 56 (46-87) | 71 (53-88) | 14:68 | 0.4940 |
| Na (mEq/L) | 138 (134-141) | 136 (132-139) | 14:68 | 0.2237 |
| Hemoglobin A1c (%) | 4.9 (4.3-5.6) | 4.9 (4.4-5.4) | 14:67 | 0.9900 |
| C-reactive protein (mg/dL) | 0.7 (0.1-1.5) | 0.4 (0.1-1.0) | 14:68 | 0.7346 |
| Procalcitonin (ng/mL) | 0.3 (0.2-0.4) | 0.2 (0.1-0.3) | 14:67 | 0.1637 |
| Prothrombin time (second) | 52 (43-66) | 50 (39-60) | 14:68 | 0.7579 |
| Prothrombin time (INR) | 1.5 (1.3-1.7) | 1.5 (1.3-1.8) | 14:68 | 0.7393 |
| Activated partial thromboplastin time (second) | 45 (40-54) | 45 (40-58) | 14:68 | 0.9362 |
| NH3 (μg/dL) | 82 (63-111) | 76 (46-111) | 14:68 | 0.4266 |
| CEA (ng/mL) | 6.5 (3.5-10.2) | 3.9 (2.4-5.4) | 13:66 | 0.1110 |
| CA 19-9 (U/mL) | 42 (13-79) | 27 (16-66) | 13:66 | 0.8844 |
| Alpha-fetoprotein (ng/mL) | 5.9 (3.0-29.9) | 6.7 (2.4-24.1) | 14:68 | 0.8149 |
| PIVKA-II (mAU/mL) | 72 (38-418) | 109 (37-432) | 14:68 | 0.8582 |
| Total cholesterol (mg/dL) | 113 (91-132) | 118 (82-151) | 6:36 | 0.8715 |
| HDLC (mg/dL) | 14 (8-33) | 30 (7-41) | 6:36 | 0.6791 |
| LDLC (mg/dL) | 49 (47-61) | 51 (26-80) | 6:36 | 0.8715 |
| Triglyceride (mg/dL) | 67 (45-102) | 60 (43-95) | 6:36 | 0.8857 |
| IgM (mg/dL) | 193 (144-248) | 148 (87-208) | 6:31 | 0.1943 |
| IgA (mg/dL) | 664 (547-792) | 493 (339-730) | 6:31 | 0.2486 |
| IgG (mg/dL) | 2117 (1522-2231) | 2151 (1749-2566) | 13:64 | 0.1804 |
| IgG at 1 week (mg/dL) | 1123 (831-1227) | 941 (730-1185) | 14:68 | 0.3035 |
| IgG at 2 weeks (mg/dL) | 752 (691-1099) | 745 (580-988) | 14:66 | 0.7040 |
| IgG at 4 weeks (mg/dL) | 946 (511-1076) | 867 (685-1112) | 12:63 | 0.6911 |
| C3 (mg/dL) | 63 (45-79) | 63 (40-92) | 12:54 | 0.9139 |
| C3 at 1 week (mg/dL) | 67 (48-76) | 63 (49-75) | 14:65 | 0.9029 |
| C3 at 2 weeks (mg/dL) | 72 (48-96) | 87 (69-113) | 14:61 | 0.0771 |
| C3 at 4 weeks (mg/dL) | 97 (69-130) | 118 (90-138) | 11:56 | 0.2042 |
| C4 (mg/dL) | 11 (7-16) | 10 (8-13) | 12:54 | 0.5822 |
| C4 at 1 week (mg/dL) | 12 (9-15) | 11 (8-14) | 14:65 | 0.6523 |
| C4 at 2 weeks (mg/dL) | 14 (10-18) | 17 (12-22) | 14:61 | 0.2554 |
| C4 at 4 weeks (mg/dL) | 21 (14-25) | 23 (17-27) | 11:56 | 0.2707 |
| Intravenous immunoglobulin | 6 (43) | 19 (28) | 14:68 | 0.3411 |
| Plasmapheresis | 0 (0) | 3 (4) | 14:68 | 1.0000 |
| Fresh frozen plasma by day 90 | 14 (100) | 57 (84) | 14:68 | 0.1969 |
| CMV by day 90 | 4 (29) | 23 (34) | 14:68 | 1.0000 |
| Infection by day 7 | 13 (93) | 23 (34) | 14:68 | 0.0001** |
| Infection by day 14 | 13 (93) | 35 (51) | 14:68 | 0.0058** |
| Infection by day 28 | 13 (93) | 38 (56) | 14:68 | 0.0132** |
| Infection by day 90 | 14 (100) | 40 (59) | 14:68 | 0.0017** |
| Early allograft dysfunction | 9 (64) | 17 (26) | 14:68 | 0.0221 |
| Acute cellular rejection by day 14 | 1 (7) | 2 (3) | 14:68 | 0.4341 |
| Death by day 90 | 5 (36) | 4 (6) | 14:68 | 0.0061** |

*Available cases (With bacteremia : Without bacteremia), **<0.05.

**Abbreviations:** IQR, interquartile range, GW/SLV, graft volume/standard liver volume; MMF, mycophenolate mofetil; DD-reconstruction, duct-to-duct reconstruction; HCC, hepatocellular carcinoma; HBV, hepatitis B virus; HCV, hepatitis C virus; HLA, human leukocyte antigen; ICU, intensive care unit; AST, aspartate transaminase; ALT, alanine aminotransferase; ALP, alkaline phosphatase; UN, urea nitrogen; GFR, glomerular filtration rate; INR, international normalized ratio; CEA, carcinoembryonic antigen; CA 19-9, carbohydrate antigen; PIVKA-II, protein induced by vitamin K absence or antagonist-II; HDLC, high-density lipoprotein cholesterol; LDLC, low-density lipoprotein cholesterol; Ig, immunoglobulin; CMV, cytomegalovirus.
